# Identification of TCR repertoires in functionally competent cytotoxic T cells cross-reactive to SARS-CoV-2

**DOI:** 10.1038/s42003-021-02885-6

**Published:** 2021-12-02

**Authors:** Kanako Shimizu, Tomonori Iyoda, An Sanpei, Hiroshi Nakazato, Masahiro Okada, Shogo Ueda, Miyuki Kato-Murayama, Kazutaka Murayama, Mikako Shirouzu, Naoko Harada, Michihiro Hidaka, Shin-ichiro Fujii

**Affiliations:** 1 Laboratory for Immunotherapy, RIKEN Center for Integrative Medical Science (IMS), Yokohama, Japan; 2 Laboratory for Protein Functional and Structural Biology, RIKEN Center for Biosystems Dynamics Research, Yokohama, Japan; 3 Division of Biomedical Measurements and Diagnostics, Graduate School of Biomedical Engineering, Tohoku University, Sendai, Japan; 4 Department of Hematology, National Hospital Organization Kumamoto Medical Center, Kumamoto, Japan; 5 Program for Drug Discovery and Medical Technology Platforms (DMP), RIKEN, Yokohama, Japan

**Keywords:** Viral infection, Immunological memory

## Abstract

SARS-CoV-2-specific CD8^+^ T cells are scarce but detectable in unexposed healthy donors (UHDs). It remains unclear whether pre-existing human coronavirus (HCoV)-specific CD8^+^ T cells are converted to functionally competent T cells cross-reactive to SARS-CoV-2. Here, we identified the HLA-A24-high binding, immunodominant epitopes in SARS-CoV-2 spike region that can be recognized by seasonal coronavirus-specific CD8^+^ T cells from HLA-A24^+^ UHDs. Cross-reactive CD8^+^ T cells were clearly reduced in patients with hematological malignancy, who are usually immunosuppressed, compared to those in UHDs. Furthermore, we showed that CD8^+^ T cells in response to a selected dominant epitope display multifunctionality and cross-functionality across HCoVs in HLA-A24^+^ donors. Cross-reactivity of T-cell receptors isolated from them exhibited selective diversity at the single-cell level. Taken together, when stimulated well by immunodominant epitopes, selective pre-existing CD8^+^ T cells with high functional avidity may be cross-reactive against SARS-CoV-2.

## Abstract (web summary)

Kanako Shimizu et al. identify HLA-A24 high-binding epitopes in the SARS-CoV-2 spike region and investigate their cross-reactivity with CD8^+^ T cell receptors. These results provide further insight into CD8^+^ T cell cross-reactivity toward SARS-CoV-2 and may be useful in future strategies for vaccine development.

## Introduction

Severe acute respiratory syndrome coronavirus 2 (SARS-CoV-2) is responsible for causing the COVID-19 pandemic. Recently, several COVID-19 vaccines, such as mRNA-based vaccines or adenovirus-vectored vaccines expressing SARS-CoV-2 spike (S) protein, have now been administered through global vaccination programs^1–3^. Neutralizing antibodies (Abs) need to be generated to protect the body by the SARS-CoV-2 vaccine. Furthermore, to inhibit SARS-CoV-2 more efficiently, CD4^+^ T cells and CD8^+^ T cells play important roles in combating SARS-CoV-2^4^. Recent studies on humoral immunity induced by the vaccines provide evidence regarding the duration of the neutralizing antibody response^5^. Moreover, it is known that CD4^+^ T cells and CD8^+^ T cells, in addition to neutralizing Abs, can play an important role in inhibiting SARS-CoV-2^4^. Anti-viral CD8^+^ cytotoxic T cells have the potential to eliminate virus-infected cells, even during the latency of viruses, such as HIV, CMV, EBV, and HSV^6–8^. Understanding anti-viral CD8^+^ T cell immunity may be useful in identifying biomarkers to evaluate the severity of or develop treatment strategies against COVID-19.

Immunodominant epitopes from structural proteins, such as S, membrane, and nucleocapsid proteins, or non-structural proteins, such as NSP7 and NSP13 encoded on ORF1 of SARS-CoV-2, have been predicted and detected in infected patients and convalescent individuals^9–16^. CD8^+^ T cell responses may be associated with disease severity^17^. A recent report demonstrated that SARS-CoV-2 specific memory CD8^+^ T cells can be detected over 6 months after the onset of symptoms in 50–70% of the convalescent patients, and their responding epitopes are present on S, membrane, nucleocapsid, and ORF3a^18^. Numerous studies of infected patients or convalescents that traced SARS-CoV-2-specific CD8^+^ T cells have demonstrated the importance of active effector or memory CD8^+^ T cells^9–16,18^.

Pre-existing CD8^+^ T cells for seasonal coronaviruses that cross-react with SARS-CoV-2 can be detected in only about 20% of the unexposed healthy donors (UHDs)^11^, but may still be biologically relevant^16^. In UHDs, the majority of immunodominant epitopes are derived from non-S regions, ORF1a, and nucleocapsid, but not S protein regions^11,19^ because of the low homology of amino acids in S protein. The cross-reactivity of CD8^+^ T cells for S protein has not been studied sufficiently. The identification of cross-reactive immunodominant epitopes in S protein in UHDs is among the key challenges for CD8^+^ T cell-induced vaccine development. In addition, as TCR cross-reactivity under specific HLA-restriction has not been studied well, molecular analysis of CD8^+^ T cell cross-reactivity is required for understanding their roles.

Patients with hematological malignancies (HM) are vulnerable to infectious diseases, including SARS-CoV-2 infections, and exhibit higher mortality than those with solid tumors^20–22^. Not only the high risk but also the worse outcomes of infection observed for HM patients are likely due to the fact that they are severely immunocompromised by the underlying hematological malignancy and systemic chemotherapy. In contrast, despite extremely low B-cell or antibody production, a sufficient CD8^+^ T cell count can reduce the COVID-19-related mortality in patients with HM^17^. Therefore, a vaccine should engage in as many facets of the immune system as possible, such as CD8^+^ T cells and antibodies. Here, we explored the immunodominant epitope on S protein for the generation of SARS-CoV-2 cross-reactive CD8^+^ T cells under HLA-A24 restriction. Using this epitope, we showed the cross-functionality of the epitope-specific CD8^+^ T cells in UHDs. We examined the mechanism underlying the cross-reactivity to seasonal coronaviruses at the single-cell TCR level. In addition, we found the decrease of these SARS-CoV-2 cross-reactive CD8^+^ T cells in the patients with HM to that in UHDs

## Results

### Identification of the immunodominant epitope in SARS-CoV-2 S in HLA-A24^+^ UHDs

**Fig. 1:**
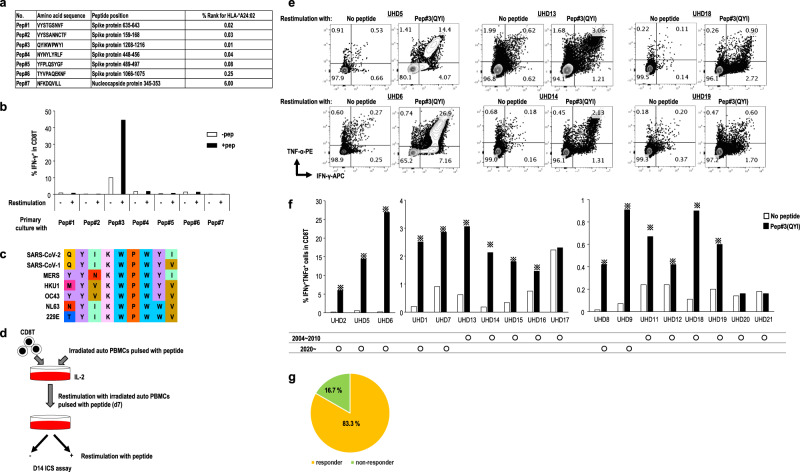
SARS-CoV-2-specific CD8^+^ T cell response in HLA-A24-positive unexposed healthy donors (UHDs). **a** Selected peptides for CD8^+^ T cells. Peptides derived from Spike (S) protein of SARS-CoV-2, shown in the list, were selected based on high-affinity binding (% rank) to HLA-A*24:02 via in silico prediction using NetMHC4.0. Pep#7 for nucleocapsid protein was selected as the control. **b** The data of IFN-γ^+^ response of antigen-specific CD8^+^ T cells from one representative donor (UHD2). PBMCs were stimulated with seven types of peptides individually, cultured for 3 weeks in the presence of IL-2, and then assessed for IFN-γ production following recall with their cognate peptide by intracellular cytokine analysis (ICS). **c** Homology of the reported epitope in the Pep#3 region of SARS-CoV-2 compared with that in other human coronaviruses. Identical residues are same color-coded. **d** Culture protocol of Pep#3(QYI)-specific CD8^+^ T cell response. The sorted CD8^+^ T cells from PBMCs of HLA-A24^+^ UHDs were stimulated with irradiated autologous PBMCs pulsed with Pep#3(QYI). The Pep#3(QYI)-specific CD8^+^ T cells were examined by the frequency of IFN-γ^+^TNF-α^+^ CD8^+^ T cells by ICS analysis on day 14. **e** Flow cytometry data are shown for representative donors (UHD5, 6, 13, 14, 18, and 19) (**e**) and the frequency of IFN-γ^+^TNF-α^+^ CD8^+^ T cells is summarized (*n* = 18) (**f**). ※ responder (>1.5 fold change (restimulation+/restimulation−) **g** Pie chart representing the proportions of responders or non-responders against Pep#3(QYI) in 18 HLA-A24^+^ UHDs.

The relationship between HLA alleles and COVID-19 has recently been reported^23^. We, therefore, assessed the interaction between HLA-A*24:02, as one of the major HLAs, and CD8^+^ T cells cross-reactive for SARS-CoV-2. Candidate targets for the CD8^+^ T immunodominant epitope in SARS-CoV-2 spike protein (SARS-CoV-2 S) were predicted using NetMHC 4.0. We selected and synthesized six candidate peptides for these epitopes from the entire S protein sequence and one epitope from nucleocapsid protein as a control (Fig. 1a)^9^.

In the current study, we used the PBMCs from healthy volunteers that were collected in early 2020 (from February to April in 2020) and during 2004–2010 (pre-pandemic era) as UHDs (Supplementary Table 1). None of the volunteers suffered from COVID-19, and they were never in close contact with a COVID-19 patient. In addition, all UHDs neither had a history of SARS-CoV-1 nor MERS infections. In addition, we assessed their plasma antibody production twice using two types of commercial kits that were often used in the previous studies^16,19,24^. All UHDs tested negative for antibodies against SARS-CoV-2. For initial screening of the optimal peptide for SARS-CoV-2 CD8^+^ T cell responses, five HLA-A24^+^ UHDs were tested. Short-term assays, such as 24-h activation induced marker assays described in the previous reports^11,13,15^, are useful for the detection of effector CD8^+^ T cells, whereas in-vitro expansion assays can be used to detect pre-existing CD8^+^ T cells against cancer or cross-reactive CD8^+^ T cells to SARS-CoV-2, even at low levels^14,25–28^. PBMCs were cultured in the presence of each peptide, and the frequencies of epitope-specific IFN-γ-producing CD8^+^ T cells were assessed on day 21. Although these six peptides from S protein were expected to bind to HLA-A*24:02 with high affinity, five of these did not induce a T-cell response, and only Pep#3(QYI)-specific CD8^+^ T cell counts were elevated in cells derived from all UHDs (Fig. 1b and Supplementary Fig. 1a, b). As positive response, we defined a 1.5-fold increase in T-cell response as compared to that in the controls (Supplementary Fig. 1c). Intriguingly, Pep#3(QYI) exhibited high sequence homology with other coronaviruses, including four types of seasonal coronaviruses (Fig. 1c). Although all the six peptides of S protein were predicted to exhibit high affinity to HLA- A*24:02, five peptides were not homologous to other seasonal coronaviruses (Supplementary Fig. 1d). These results suggest that CD8^+^ T cells may be cross-reacting, and that pre-existing memory CD8^+^ T cells may be responsible for the immune response.

We then focused on Pep#3(QYI)-specific CD8^+^ T cells from UHDs. In this assay, we isolated CD8^+^ T cells from PBMCs and cocultured them with irradiated autologous PBMCs pulsed with Pep#3(QYI), and the frequencies of epitope-specific IFN-γ-producing CD8^+^ T cells were assessed on day 14 (Fig. 1d). We found that antigen-specific CD8^+^ T cells were present in 15 out of 18 UHD participants (83.3%) (Fig. 1e, f, g). The response rate of Pep#3(QYI)-specific T cells in HLA-A*24:02^+^ UHDs was higher than that in 20% of the UHDs in a previous study^11^. Even if the frequency was low, most of the responders possessed IFN-γ-and TNF-α-producing T cells (Fig. 1e, f).

### Assessment of the polyfunctionality of Pep#3(QYI)-specific CD8^+^ T cells from UHDs

**Fig. 2:**
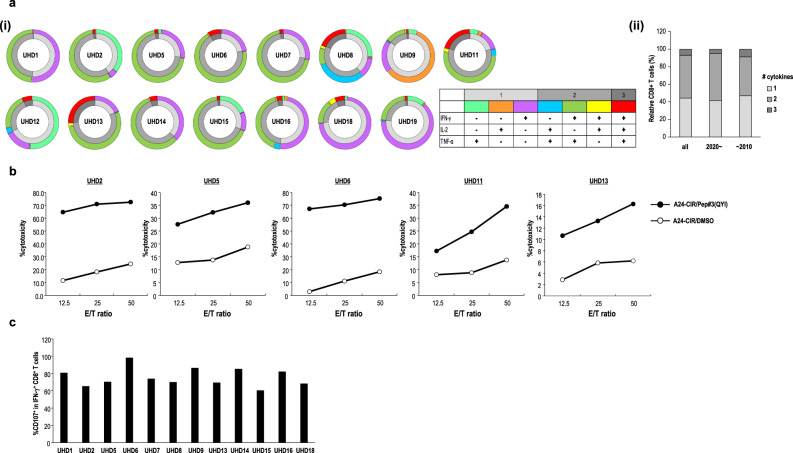
Function of SARS-CoV-2-specific CD8^+^ T cells from UHDs. **a** Polyfunctionality of SARS-CoV-2 Pep#3(QYI)-specific CD8^+^ T cells. Pep#3(QYI)-specific CD8^+^ T cell lines were established from UHDs and re-stimulated individually with Pep#3(QYI) in an ICS assay (*n* = 15). The frequency of CD8^+^ T cells with different cytokine production profiles (IFN-γ, TNF-α, and IL-2) was determined. (i) The outer ring of the double ring pie shows the cytokine profile in different colors, and the inner ring represents the number of cytokines produced, with gray scale. (ii) The mean of the frequency of number of cytokines produced in UHDs (early 2020) and UHDs (pre-pandemic 2004–2010) and all was summarized. **b** Peptide-specific cytotoxic activity of Pep#3(QYI)-specific CD8^+^ T cells from representative donors. The cytotoxicity assay was performed in duplicate. Data represent the mean of duplicate. **c** Frequency of the degranulation marker CD107a positive in Pep#3(QYI)-specific IFN-γ^+^ CD8^+^ T cells (*n* = 12).

We assessed the polyfunctionality of Pep#3(QYI)-specific CD8^+^ T cells by cytokine production capacity, cytotoxic activity, and degranulation markers. First, we found that half the T-cell populations produced more than two cytokines (IFN-γ, TNF-α, and IL-2) (Fig. 2a). In contrast, we could not detect IL-10 (Supplementary Fig. 2). Next, the cytotoxicity of Pep#3(QYI)-specific CD8^+^ T cell lines was assessed (Fig. 2b). We detected cytotoxicity against Pep#3(QYI)-pulsed A24/CIR, but not the unpulsed A24/CIR (Fig. 2b). Finally, we performed the CD107a degranulation assay, which is used to measure the potential of cells to secrete cytotoxic molecules, i.e., perforin and granzyme. We found that approximately 80 % of IFN-γ^+^ CD8^+^ T cells expressed CD107a (Fig. 2c).

### Detection of the high affinity region around the Pep#3(QYI)

**Fig. 3:**
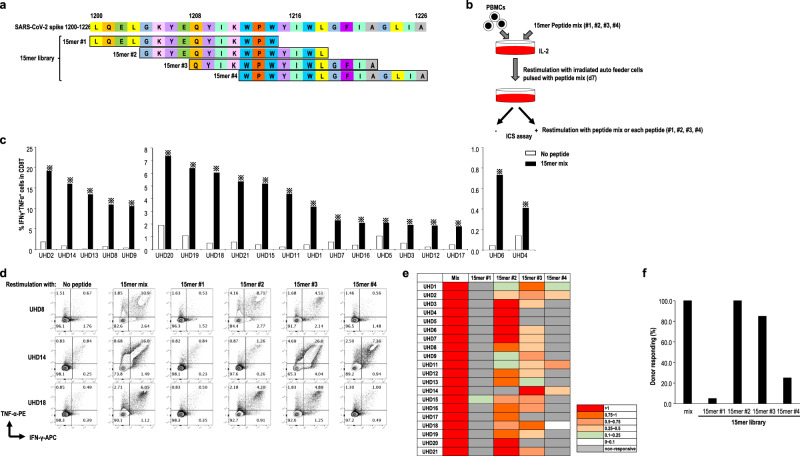
Design of the 15-mer peptide mix-responding CD8^+^ T cells. **a** Design of 15-mer peptide library, including Pep#3(QYI) epitopes, in the S2 region of SARS-CoV-2. **b** Analysis of the 15-mer peptide-responding CD8^+^ T cells. PBMCs from UHDs were cultured in the presence of a 15-mer peptide mix. Two weeks later, the cultured cells were restimulated with or without the peptide mix for 16 h in the presence of monensin and brefeldin. The frequency of IFN-γ^+^TNF-α^+^ CD8^+^ T cells was measured after gating the CD8^+^ T cells by an ICS assay at day 14. **c** Data show the frequency of IFN-γ^+^TNF-α^+^ CD8^+^ T cell response to the 15-mer peptide mix in UHDs (*n* = 20). ※ responder (>1.5 fold change (restimulation +/restimulation−). **d** As shown in (**b**), the cultured cells were restimulated with or without the peptide mix or each 15-mer peptide for 16 h in the presence of monensin and brefeldin. The frequency of IFN-γ^+^TNF-α^+^ CD8^+^ T cells was measured after gating the CD8^+^ T cells by an ICS assay. Flow cytometry data from representative donors. **e** Summary of the 15-mer peptide-responding CD8^+^ T cells. The peptide-specific CD8^+^ T cell frequency was calculated by subtracting the frequency of IFN-γ^+^TNF-α^+^ CD8^+^ T cells cultured without peptide restimulation (CD8T (pep−)) from the frequency of IFN-γ^+^TNF-α^+^ CD8^+^ T cells cultured with peptide restimulation (CD8T (pep+)). Data indicate the ratio of the frequency of each 15-mer peptide-responding IFN-γ^+^TNF-α^+^ CD8^+^ T cells to that of the 15-mer peptide mix responding IFN-γ^+^TNF-α^+^ CD8^+^ T cells (*n* = 20). **f** Frequency of donors responding to each 15-mer peptide.

Next, we synthesized four types of 11 mer-overlapped 15-mer peptides based on the S2 region (1200-1226) of S protein around the Pep#3(QYI) epitope, to examine the other immunodominant epitopes in this region (Fig. 3a). Since the relevant epitopes are relatively conserved across HCoVs and completely conserved across the SARS-CoV-2 variant strains, such as VOC-202012/01, 501Y.V2, and 501Y.V3, the peptide set is available for SARS-CoV-2 variant strains (Supplementary Fig. 3). We cultured PBMCs from UHDs in the presence of a 15-mer peptide mix and evaluated the response of antigen-specific CD8^+^ T cells at day 14 (Fig. 3b). Surprisingly, we detected SARS-CoV-2-specific CD8^+^ T cells in all the UHDs exposed to the peptides (Fig. 3c). Moreover, CD8^+^ T cells responded primarily to 15-mer pep#2 and #3, but in some cases, they also responded to 15-mer pep#1 and #4 (Fig. 3d–f). These findings indicated that the 15-mer peptide mix contained the other immunodominant epitopes in addition to Pep#3(QYI) for HLA-A24^+^ individuals and would be more useful for detecting or expanding pre-existing CD8^+^ T cells in HLA-A24^+^ individuals.

### Multiple epitopes in SARS-CoV-2 S are highly immunogenic in HLA-A24^+^UHDs

**Fig. 4:**
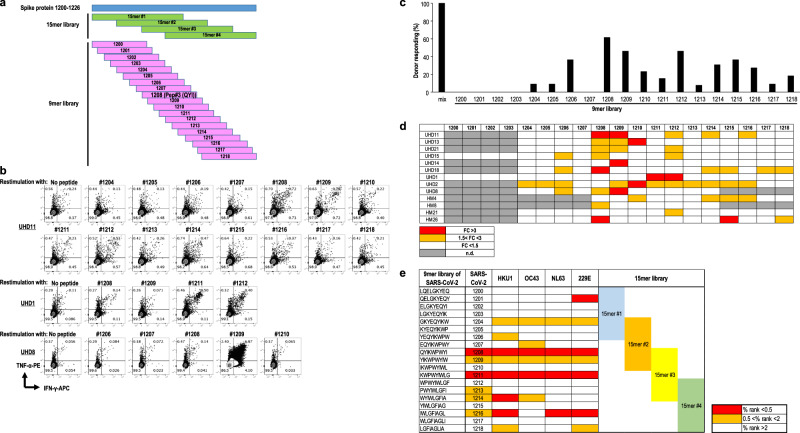
Epitope mapping in spanning 15-mer peptide mix. **a** Design of 9-mer peptide library in S protein_1200–1226_. **b** Flow cytometry data from representative donors (UHD 1, 8, and 11) are shown. **c** Frequency of donors responding to each peptide of the 9-mer peptide library. **d** Response to 9-mer peptide-specific CD8^+^ T cells selected from the 15-mer peptide mix. Data indicate that the fold change (FC) of CD8^+^ T (pep+) to CD8^+^ T (pep−) was 1.5 times higher (orange) and 3 times higher (red) in the 9-mer peptide, respectively. (*n* = 12) Gray indicates not done. **e** In-silico analysis of the affinity for HLA-A24 in both SARS-CoV-2 S protein_1200–1226_ and the relevant region of seasonal coronaviruses using IEBD and NetMHC4.0. (%rank < 0.5 (red), 0.5 < %rank < 2 (orange), %rank > 2 (white)).

Next, to verify the parts of the epitopes of four types of the 15-mer peptide mix responsible for CD8^+^ T cell activation, a consecutive 9-mer peptide library was synthesized using a S protein sequence (Fig. 4a). We assessed peptide-specific T-cell response by restimulation with each 9-mer-peptide after priming with the 15-mer peptide mix (Fig. 4b). We found that the 9-mer peptides#1204 to #1218, particularly #1208 (Pep#3(QYI)), induced CD8^+^ T cell-specific responses comparable to 15-mer pep#2 to #3 (Fig. 4c, d). Accordingly, we conducted *an* in-silico screening of this region in SARS-CoV-2 and seasonal coronaviruses using IEBD and NetMHC4.0 (Fig. 4e) to examine which 9-mer peptides were recognized by these CD8^+^ T cells under the HLA-A24 restriction. We found six and eight types of 9-mer-epitopes with high IEBD and NetMHC scores expressed on SARS-CoV-2 as well as seasonal coronaviruses, which suggested that they bind to HLA-A24 (Fig. 4e). Thus, we found that some of the 9-mer peptides identified in our in-vitro experiments were the high affinity peptides predicted in silico under HLA-A24 restriction. However, other peptides may bind to other HLAs.

### Analysis of SARS-CoV-2-specific CD8^+^ T cells in patients with HM

**Fig. 5:**
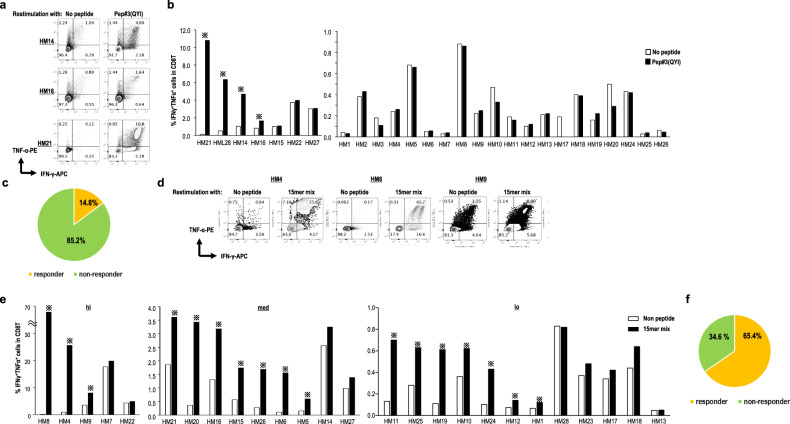
SARS-CoV-2-specific CD8^+^ T cell response in HLA-A*24:02-positive patients with hematological malignancy (HM). **a**–**c** Sorted CD8^+^ T cells from HLA-A*24:02-positive patients with HM were cultured with irradiated autologous PBMCs pulsed with SARS-CoV-2 Pep#3(QYI). The peptide-specific CD8^+^ T cells were assessed by the frequency of IFN-γ^+^TNF-α^+^ CD8^+^ T cells by an ICS assay on day 14. Flow cytometry data are from representative donors (HM14, 16, and 21) (**a**). The frequency of IFN-γ^+^TNF-α^+^ CD8^+^ T cells is summarized (*n* = 27) (**b**). Pie chart representing the proportions of responders or non-responders against Pep#3(QYI) in patients with HM (**c**)**. d**–**f** PBMCs from patients with HM were cultured in the presence of a 15-mer peptide mix. Two weeks later, the cultured cells were restimulated with or without the mix for 16 h in the presence of monensin and brefeldin. The frequency of IFN-γ^+^TNF-α^+^ CD8^+^ T cells was measured after gating the CD8^+^ T cells by an ICS assay at day 14. Flow cytometry data are from representative donors (HM4, 8, and 9) (**d**). The frequency of IFN-γ^+^TNF-α^+^ CD8^+^ T cells is summarized (*n* = 26) (**e**). ※responder (>1.5 fold change (restimulation+/restimulation−). Pie chart representing the proportions of responders or non-responders against the 15-mer peptide mix in patients with HM (**f**).

We examined whether Pep#3(QYI) can induce epitope-specific CD8^+^ T cells in HLA-A24^+^ patients with HM (Supplementary Table 2), who represent a high-risk group that may be susceptible to COVID-19 due to immune-cell dysfunction related to disease progression or chemotherapy. We confirmed that all patients with HM recruited in this study were negative for IgG specific for SARS-CoV-2 (Supplementary Table 2). Pep#3(QYI)-specific CD8^+^ T cells were observed in 14.8% (4 out of 27) of the patients with HM, and the induction ratio of Pep#3(QYI)-specific CD8^+^ T cells was significantly lower in patients with HM than in UHDs (*p* < 0.01; Fisher’s/Chi-square tests) (Fig. 5a–c). This indicated that the levels of SARS-CoV-2 cross-reacting CD8^+^ T cells against seasonal coronaviruses were lower in patients with HM than in UHDs.

Next, we analyzed these patients by four types of 11 mer-overlapped 15-mer peptides (Fig. 5d–f). Surprisingly, we detected SARS-CoV-2-specific CD8^+^ T cells in 65.4% of patients with HM (Fig. 5e, f). T-cell response in the presence of a mix of four peptides was more efficient than that induced by the 9-mer peptide, particularly in patients with HM. Thus, there were fewer cross-reactive CD8^+^ T cells in patients with HM than in UHDs (65.4% vs 14.8%, *p* < 0.001; Fisher’s/Chi-square tests), which indicated that seasonal coronavirus-specific memory T cells may be reduced in response to disease progression or therapy-related toxicity. However, CD8^+^ T cells that have the potential to respond to the 15-mer mix, mainly to 15-mer pep#2 and #3, can be activated by rechallenging with the 15-mer peptide mix. Furthermore, in some cases, they also responded to 15-mer pep#1 and #4 (Supplementary Fig. 4).

### CD8^+^ T cells exhibit cross-reactivity between SARS-CoV-2- and HCoV-derived S peptide

**Fig. 6:**
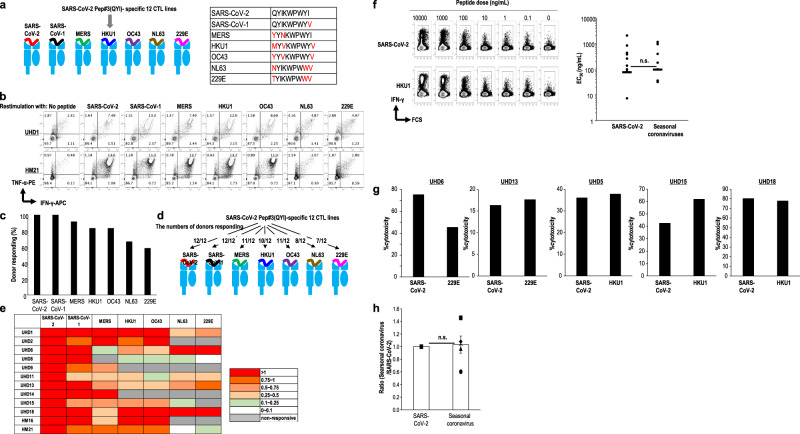
Cross-reactivity of SARS-CoV-2 Pep#3(QYI)-specific CD8^+^ T cells to other coronaviruses. **a** Cross-reactivity of SARS-CoV-2 Pep#3(QYI)-specific CD8^+^ T cell lines from 12 UHDs and patients with HM to the relevant peptides of other coronaviruses (SARS-CoV-1, MERS, HKU1, OC43, NL63, and 229E). CD8^+^ T cell lines were restimulated with the indicated peptide or DMSO for 16 h and analyzed using IFN-γ-APC and TNF-α-PE by ICS analysis. **b** Representative flow cytometry data from UHD1 and HM21 are shown. **c** Frequency of donors responding to each peptide of the coronaviruses. **d** The numbers of donors responding to each peptide of the coronaviruses. **e** The peptide-specific CD8^+^ T cell frequency was calculated by subtracting the frequency of CD8^+^ T (pep−) from that of CD8^+^ T(pep+). The ratio of each coronavirus-derived peptide-specific IFN-γ^+^TNF-α^+^ CD8^+^ T cell frequency to that of CoV-2 pep#3 is depicted. (*n* = 12). **f** Avidity of antigen-specific CD8^+^ T cells. Flow cytometry data show IFN-γ expression for the indicated concentrations of peptides from SARS-CoV-2 and seasonal coronavirus (UHD1). Based on these data, half maximal response (EC_50_) of Pep#3(QYI)-specific CD8^+^ T cells was measured in the response to cognate peptide and the relevant peptide of seasonal coronaviruses. (*n* = 9) (n.s., Mann–Whitney *U*-test). **g**, **h** Cross-functionality of Pep#3(QYI)-specific CD8^+^ T cells. Peptide or -specific cytotoxic activity of Pep#3(QYI)-specific CD8^+^ T cells from 5 UHDs (UHD5, 6, 13, 15, and 18) was compared. Cytotoxic T cell assays were performed using Pep#3(QYI)-pulsed A24/CIR or the seasonal coronavirus derived peptide-pulsed A24/CIR as target cells (E/T ratio = 50). The cytotoxicity assay was performed in duplicates. Data represent the mean of duplicate (**g**). The white bar and other symbols represent the mean of values from five UHDs and each data, respectively. (mean ± SEM) (n.s., Mann–Whitney *U*-test) (**h**).

We evaluated whether the Pep#3(QYI)-specific CD8^+^ T cells generated from ten HLA-A24^+^ UHDs and two patients with HM cross-reacted with other coronavirus families, and quantified SARS-CoV-2-specific CD8^+^ T cells (Fig. 6a). The Pep#3(QYI)-specific CD8^+^ T cell lines were assessed based on the production of IFN-γ and TNF-α in response to the relevant peptide of each virus (Fig. 6b). The Pep#3(QYI)-specific CD8^+^ T cell lines from UHDs exhibited cross-reactivity across the HCoVs. In these two patients with HM, cross-reactivity of CD8^+^ T cells was also found to be preserved. The CD8^+^ T cell lines responded to betacoronaviruses (SARS-CoV-1, MERS, HKU-1, and OC43), with 83–100% responders, and to alphacoronaviruses (NL63 and 229E), with 58.3–66.7% responders (Fig. 6c–e).

Next, we assessed the level of the cross-reactivity of Pep#3(QYI)-specific CD8^+^ T cells to coronaviruses in UHDs. We also evaluated the functional avidities of these CD8^+^ T cells by IFN-γ production. Peptide titration experiments defined a dose range of 0 to 10,000 ng/mL for SARS-CoV-2 and seasonal coronavirus with the highest affinity (e.g., HCoV-HKU1) (Fig. 6f). EC_50_ is calculated as the peptide concentration required to reach one-half maximal response. EC_50_ for SARS-CoV-2 S was comparable to that for seasonal coronaviruses (Fig. 6f). These data demonstrated similar functional avidity of the SARS-CoV-2 cross-reactive CD8^+^ T cell response relative to the CD8^+^ T cell response for seasonal coronaviruses. Furthermore, we assessed the cytotoxic activity across different coronaviruses as another measure of cross functionality (Fig. 6g, h). The cytotoxicity against A24/CIR pulsed with the target peptide from SARS-CoV-2 was compared to that from seasonal coronaviruses with the highest affinity for Pep#3(QYI)-specific CD8^+^ T cells in each UHD cell line (Fig. 6g). However, no statistical difference was observed between them (Fig. 6h). These findings indicated that although some pre-existing memory CD8^+^ T cells in HLA-A24^+^ UHDs have the potential to recognize SARS-CoV-2, they can be sufficiently skewed toward SARS-CoV-2 under optimal conditions.

### TCR avidity for recognition of SARS-CoV-2 and HCoVs at the single-cell level

**Fig. 7:**
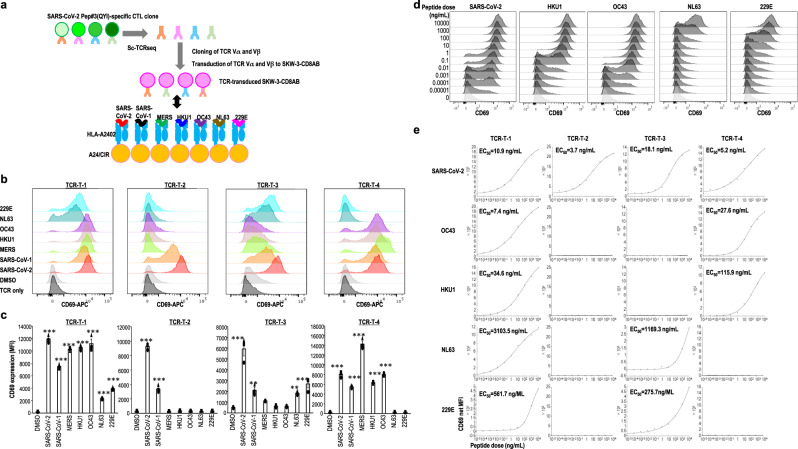
Cross-reactivity of cloned TCRs across coronaviruses. **a** Cross-reactivity of cloned TCRs. To obtain functional antigen-specific TCR molecules from T cell repertoires of Pep#3(QYI)-specific CD8^+^ T cell line, TCR-α/β chains were cloned from Pep#3(QYI)-specific CD8^+^ T cells, followed by functional validation of the completed TCRs. The TCR-transduced SKW3-CD8AB cells were cocultured with the indicated peptide-pulsed A24/CIR for 16 h, and the upregulation of CD69 expression was measured by flow cytometry. **b** CD69 expression of TCR-transduced SKW3-CD8AB. These data are representative of all experiments repeated three times. **c** Summary of CD69 expression (net MFI) of TCR-transduced SKW3-CD8AB cells in (**b**) is depicted. The white bar and other symbols represent the mean and each data, respectively. (mean ± SEM) **p < 0.01, ***p < 0.001 (Newman–Keuls test). **d**, **e** Avidity of Pep#3(QYI)-specific TCRs. Flow cytometry data show CD69 expression of TCR-T-1 for the indicated concentrations of peptides from SARS-CoV-2 and from seasonal coronaviruses (**d**). Based on data, half maximal response (EC_50_) of TCR-transduced SKW3-CD8AB was measured in response to cognate peptide and the relevant peptide of seasonal coronaviruses. Plot data are shown as CD69 net MFI at peptide concentration (**e**). These data are representative of all experiments repeated twice.

To understand why CD8^+^ T cells exhibit multiple responses to various coronaviruses and the underlying mechanisms, we studied the cross-reactivity of CD8^+^ T cells at the single TCR level. After restimulation with Pep#3(QYI), we isolated CD8^+^ T cells from Pep#3(QYI)-specific CD8^+^ T cell lines by gating CD107a^+^CD8^+^ cells and performed single-cell analysis of TCR repertoires. To analyze the clonalities of Pep#3(QYI)-reactive CD8^+^ T cells, a total of 227 T cells were screened, and 44 TCR clonotypes were identified from five UHDs and one patient with HM (Supplementary Fig. 5). Furthermore, we focused on the dominant TCR types from among the clonotypes and identified four types of TCRα and TCRβ pairs from four donors (three UHDs and one HM). To assess the specificity and functions of cloned TCRs, we transduced the *TCRα* and *TCRβ* genes into the SKW3-CD8AB (human T-ALL) cell line and demonstrated their peptide-specific response (Fig. 7a). Intriguingly, the four types of TCR repertoires varied from each other in their epitope recognition (Fig. 7b). TCR-T (TCR-T-1) cells from UHD2 responded well to all the epitopes derived from coronaviruses, whereas the TCR-T (TCR-T-2) cells from UHD8 responded only to SARS-CoV-1 and SARS-CoV-2. These data are interesting because UHD8 had not been infected with either of them. Dykema et al. also reported the same phenomenon in a previous study^27^, wherein SARS-CoV-2-specific, monoreactive CD4^+^ T clonotypes were detected in the UHDs and indicated the possibility of cross-reactive response to unknown coronaviruses or other pathogens. The TCR-T-2 data, however, may also be a chance finding. Furthermore, the TCR-T (TCR-T-3) cells from UHD6 responded to SARS-CoV-2, SARS-CoV-1, NL63, and 229E, while those (TCR-T-4) from HM16 responded to SARS-CoV-2, SARS-CoV-1, MERS, HKU1, and OC43, but not to NL63 and 229E (Fig. 7b, c). Some types of single TCRαβ-transduced T cells responded broadly to the relevant epitopes on HCoVs, whereas others responded only to two or four peptides. These findings on the SARS-CoV-2-epitope-responding T cell repertoires imply that TCRαβ may be selective at the single-cell level.

We next evaluated the functional avidities of these TCR-transduced T cells by upregulation of CD69 expression (MFI). Peptide titration experiments defined a dose range of 10 fg/mL to 10 μg/mL for SARS-CoV-2 and each seasonal coronavirus (Fig. 7d). TCR-T-1 exhibited the highest avidity for OC43 compared to that for SARS-CoV-2 and others. However, other TCR-T cells (TCR-T-2 to TCR-T-4) from UHDs exhibited the highest avidity for SARS-Co-V-2 (Fig. 7e). Comparing TCR-T-2 (monoreactive) to other cross-reactive TCRs (TCR-T-1, -3, and -4), we could not find large differences in their functional avidities (Fig. 7e). Irrespective of the previous infection status, Pep#3(QYI)-specific CD8^+^ T cells from UHDs displayed sufficient cross-functionality at the single cell level (Fig. 7e). From these data, we concluded that Pep#3(QYI)-specific CD8^+^T cells from UHDs did not exhibit low avidity.

### Crystal structures of CD8^+^ T cells binding to HLA-A*24:02 and TCR complex

**Fig. 8:**
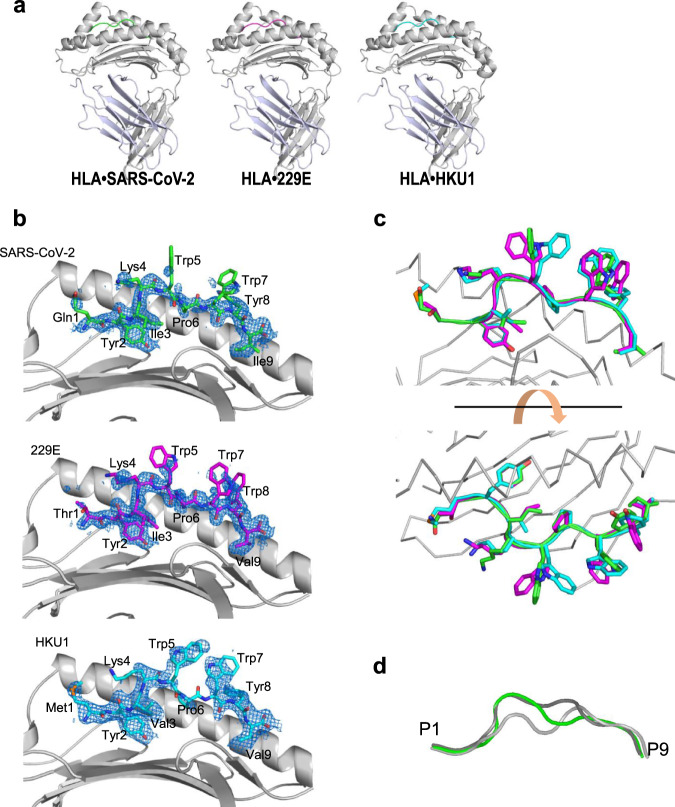
Structure of the peptide-binding site. **a** Crystal structures of three HLA-A*24:02•peptide complexes. HLA-A24 structures are drawn as ribbon models in gray/light purple, and peptide structures are colored in green (CoV2), magenta (229E), and cyan (HKU1). **b** Electron densities of the peptides. The electron density for each peptide is contoured at 1.0σ in the 2Fo-Fc omit map. Peptide structures are shown in green (CoV2, upper panel), magenta (229E, middle panel), and cyan (HKU1, bottom panel). **c** Superimposition of the three peptide structures. Peptide structures are depicted by the same colors as in (**b**). **d** Superposition of the 9-mer peptide structures in the HLA-A*24:02•peptide complexes. Peptides are shown in green (CoV2, this study), light gray (PDB code: 2BCK), and dark gray (PDB code: 3I6L).

The crystal structures of HLA-A*24:02 and three peptides, namely, SARS-CoV-2, 229E, and HKU1, were determined (Fig. 8a, Table 1). These three complex structures were almost identical to each other and their rmsd values were 0.15 Å, 0.41 Å, and 0.41 Å between HLA-A*24:02•CoV2 and HLA-A A*24:02•229E, HLA-A*24:02•CoV2 and HLA-A*24:02•HKU1, and HLA-A*24:02•229E and HLA- A*24:02•HKU1, respectively. In the complex structures, crystal packing was not involved in the interactions between HLA- A*24:02 and each peptide.

**Table 1:** Data collection and refinement statistics.

	HLA-A*24:02•CoV2	HLA-A*24:02•229E	HLA-A*24:02•HKU1
*Data collection*			
Space group	*P*2_1_2_1_2_1_	*P*2_1_2_1_2_1_	*P*2_1_2_1_2_1_
Cell dimensions			
* a*, *b*, *c* (Å)	67.72, 85.47, 91.37	67.95, 85.59, 91.09	66.70, 77.72, 87.95
α, β, γ (°)	90.00, 90.00, 90.00	90.00, 90.00, 90.00	90.00, 90.00, 90.00
Resolution (Å)	1.89 (1.96–1.89)*	1.71 (1.77–1.71)	2.10 (2.18–2.10)
*R*_merge_	0.220 (2.797)	0.096 (2.250)	0.136 (2.179)
*I* / σ*I*	7.7 (0.7)	13.5 (0.8)	7.7 (0.8)
Completeness (%)	98.73 (97.72)	99.99 (99.97)	99.96 (99.90)
Redundancy	7.8 (7.8)	7.7 (7.6)	7.1 (7.3)
*Refinement*			
Resolution (Å)	1.89	1.71	2.11
No. reflections	41,912	57,838	26,856
*R*_work_/*R*_free_	0.200/0.255	0.192/0.221	0.203/0.264
No. atoms			
Protein	3071	3071	3087
Ligand/ion	94	93	91
Water	378	491	136
*B*-factors			
Protein	32.1	30.3	52.9
Ligand/ion	49.6	44.3	70.2
Water	37.9	37.9	50.3
R.m.s. deviations			
Bond lengths (Å)	0.007	0.007	0.008
Bond angles (°)	0.86	0.83	0.93

*Values in parentheses are for highest-resolution shell.

The 9-mer peptides were identified at the equivalent position of the HLA-A*24:02 structure (Fig. 8b). Although electron densities of the peptide backbones were observed, those of some side chains were poor, particularly, Lys4 for three peptides, Trp5 and 7 for CoV2 and 229E, and Pro6 for HKU1. The conformation of these peptide backbones overlapped well. Of the nine positions, the side chains at positions P2, P3, P6, and P9 were involved in the HLA-A*24:02•peptide interactions at the bottom of the binding pocket. In contrast, the side chains at positions P1, P4, P5, P7, and P8 were directed to the solvent region and oriented differently in the complex structures (Fig. 8c). Thus, these side chains are expected to directly interact with TCR.

In the Protein Data Bank, 31 structures of the HLA-A*24:02•peptide complex have been deposited so far. Peptide lengths are in the range of 8-mer to 11-mer. Of these, two complex structures are with the 9-mer peptide^29^. A comparison between these 9-mer peptides and the peptides in this study revealed that all the peptides anchored to the binding pocket at positions P2 and P9, and conformational variations were observed at positions P4–P7 (Fig. 8d). This region forms a bulge structure and corresponds to the putative interaction site with TCR. Furthermore, we investigated the influence of peptide sequence on the interface and the intrinsic plasticity of the TCR/pMHC trimolecular and pMHC biomolecular complexes via structural modeling performed by replacing the HLA moiety of the HLA•peptide•TCR complex structure^30^ (PDB code 3VXM) with the structure mentioned in the present study.

## Discussion

Elucidating the role of pre-existing, cross-reactive T cells in COVID-19 is important for understanding the development and severity of COVID-19 and determining the optimal T-cell engagement strategies for development of diagnostic tools and vaccines. An expansion of cross-reactive T cells with low avidity may lead to exacerbation of infection. Therefore, it is critical not only to identify pre-existing T cells but also to find an approach to expand functionally competent, cross-reactive T cells with high avidity. In the current study, we focused on an amino acid sequence conserved between SARS-CoV-2 and seasonal coronaviruses in the S2 region, and selected 9-mer peptides. One of the selected peptides, Pep#3(QYI), efficiently induced SARS-CoV-2 cross-reactive CD8^+^ T cells in HLA-A24^+^ UHDs. These Pep#3(QYI)-specific CD8^+^ T cells from UHDs exhibited polyfunctionality and similar cytotoxicity against seasonal coronaviruses. Furthermore, we identified immunodominant epitopes, including Pep#3 (QYI), which were covered by three 15-mer overlapping peptides. Moreover, to elucidate the mechanism, we assessed the cross-reactivity and functional avidity of Pep#3 (QYI)-specific TCR at the single TCR level.

Although cross-reactivity with pre-existing T cells has been reported^10–14,19,31^, whether SARS-CoV-2 cross-reactive T cells are protective is still an issue of debate. SARS-CoV-2 cross-reactive CD4^+^ T cells have been reported to exhibit low avidity, and some studies have demonstrated that they are non-protective^24,27^. However, there are three studies that address the cross-reactivity of pre-existing CD8^+^ T cells in UHDs. Schulien et al. performed an analysis of pre-existing and induced CD8^+^ T cells in three patients pre- and post-SARS-Cov-2 infection and showed the importance of anti-viral effector CD8^+^ T cells^16^. T cell cross-reactivity can be defined to recognize more than one distinct peptide-MHC structures by single TCR^32^. Mallajosyula et al. demonstrated that T cells that recognize peptides conserved among coronaviruses are abundant in UHDs^33^. Moreover, they verified that these are more abundant in COVID-19 patients by spheromer technology. They also detected shared TCR motifs of SARS-CoV-2 specific CD8^+^ T cells between UHDs and mild COVID-19 patients^33^, suggesting a protective role of pre-existing CD8^+^ T cells in COVID-19. A recent study reported immunodominant, HLA-B7-restricted SARS-CoV-2 nucleocapsid protein epitope cross-reactive CD8^+^ T cells^28^. The authors found the presence of a shared CDR3β motif in epitope-specific CD8^+^ T clonotypes in exposed and unexposed donors, which suggested that pre-existing immunity in HLA-B7^+^ individuals favored clonal expansion. In the current study, we demonstrated the pattern of Pep#3 (QYI)-specific CD8^+^ T cell TCR cross-reactivity across HCoVs under HLA-A24*02 restriction at the single-cell level, which exhibited substantial functional avidity. Nevertheless, further molecular studies that examine TCR characteristics of HLA-A24^+^ UHDs and infected patients are required.

Cancer patients have an increased risk of severe illness from COVID-19, with a high mortality rate^20,34,35^. Particularly, adult patients with HM having COVID-19 were found to have a 34% risk of death. Patients aged >60 years had a significantly higher risk of death^22^. Furthermore, patients with impaired B-cell function, such as myeloma, lymphoma, or CLL, may not respond to standard vaccines and may require special attention^36,37^. Using a 15-mer peptide mix, SARS-CoV-2 cross-reactive CD8^+^ T cells were successfully detected at 75%, 50%, and 56% higher rates in AML, CML, and MM, respectively, compared to those detected using Pep#3(QYI). Thus, the 15-mer peptide mix may act as CD8^+^ T-cell-induced vaccines or immune monitoring tools for the efficacy of COVID-19 vaccines in high-risk HLA-A24^+^ patients with HM. Since these findings indicate the importance of HLA in COVID-19 pathogenesis, other HLA and peptide interactions should also be studied.

The restricted formula for linking HLA-peptide and TCR confers heterotypic cross-reactivity across SARS-CoV-2. Furthermore, to elucidate the molecular basis of cross-reactive TCRα/β recognition between SARS-CoV-2 and seasonal coronaviruses, we examined the structure of SARS-CoV-2 peptides and the relevant peptides of seasonal coronaviruses bound to the HLA-A*24:02 molecule (Fig. 8). The obtained data on the HLA-A*24:02•peptide complex structures imply that the side chains at positions P1, P4, P5, P7, and P8 interact with the TCR. The superimposed model (where the HLA moiety is replaced by the structure presented in our study) indicates that the peptide positions mentioned above are close to the contact surface of the TCR. The side chains at positions P1/P4/P5 and P7/P8 can interact with the loop moieties of TCRα and TCRβ, respectively. In the sequence alignment among TCR1-4, insertions/deletions were found in these loop regions, suggesting variety of affinities between the HLA-A*24:02 peptide complex and TCRs. Furthermore, our data suggest that the selective cross-reactivity of T cells in SARS-CoV-2 epitopes depends on combinations of the structure mode of the peptide on the HLA-A*24:02 complex and the sequences/loop region of the TCR conformation. Further studies are required to broadly underpin the cross-reactivity by analyzing the structure of the cross-reactive TCR complex with HLA-A*24:02•SARS-CoV-2-S2 and HLA- A*24:02•seasonal coronavirus epitopes.

Overall, we found that the potential of cross-reactivity may depend on the interaction between TCR and the structure of specific HLA, i.e., HLA- A*24:02. Moreover, TCRα/β from Pep#3(QYI)-specific CD8^+^ T cells exhibited varying patterns of cross-reactivity against HCoVs. Lastly, we determined the optimal 15 mer-mixed peptides that may stimulate SARS-CoV-2-specific CD8^+^ T cells, even for most patients with HM in addition to all UHDs; the results indicated the possibility of stimulatory epitopes. We speculated that some CD8^+^ T cells that act against seasonal coronaviruses might persist as long-term memory cells in UHDs. If these cross-reactive T cells were stimulated by a type of vaccine, including immunodominant epitopes, these could be skewed toward SARS-CoV-2. Development of a vaccine for modulating cross-reactive CD8^+^T cells may help reduce the rates of the disease.

## Methods

### Human samples and preparation

Peripheral blood samples were obtained from healthy blood donors at our Institute (early 2020) or from the buffy coats of healthy blood donors (2004–2010) (Tokyo Red Cross, Tokyo, Japan). Peripheral blood samples from 28 patients with hematological malignancies (HM) were obtained from the National Hospital Organization Kumamoto Medical Center (Kumamoto, Japan). None of the UHDs or unexposed patients with HM suffered from COVID-19, and they did not have any close contact with a COIVD-19 patient. In addition, none of the UHDs had a history of SARS-CoV-1 nor MERS infections. Plasma samples from healthy donors and patients with HM were assessed by COVID-19 Human IgM IgG Rapid tests (Abnova) and the anti-SARS-CoV-2 IgG ELISA kit (EUROIMMUN). The sensitivity of the Euroimmun kit in samples after day 10 is 94.5%, according to the manufacturer. None of the patients tested positive for either antibody. These donors are referred to as unexposed donors (UHDs) in this manuscript. PBMCs were separated via Ficoll-Paque PLUS (GE Healthcare, Uppsala, Sweden) density centrifugation and washed twice with phosphate-buffered saline (PBS) and stored in liquid nitrogen until further use. Information on healthy blood donors and the characteristics of patients with HM are listed in Supplementary Tables 1 and 2, respectively. This study was approved by the Institutional Review Board for Human Research at RIKEN IMS. Written informed consent was obtained from all healthy donors and patients according to the Declaration of Helsinki. HLA-A24^+^ donors were selected using flow cytometry for T-cell analysis.

### Cell lines

CIR cells expressing HLA-A*24:02 (A24/CIR) were kindly provided by Dr. Masafumi Takiguchi (Kumamoto University)^38^. The SKW-3 (T-ALL) line was procured from RIKEN BRC (Japan). All cell lines were tested according to the manufacturer’s protocol and proved to be mycoplasma free (Mycoplasma Detection Kit; Minerva Biolabs). A24/CIR has been routinely tested for HLA-A24 expression by our hands. According to the International Cell Line Authentication Committee register (https://iclac.org), SKW-3 cells were misidentified as KE-37 cells. We verified that our cells lack TCRs and functionally work like T cells when transduced with T cell receptors and CD8AB.

### Epitope prediction and peptide selection

The S protein CD8^+^ T epitopes of SARS-CoV-2 and other HCoV for HLA-A*24:02 were predicted using the Immune Epitope Database and Analysis Resource (IEDB) (https://www.iedb.org/)^39^ and NetMHC 4.0 (http://www.cbs.dtu.dk/services/NetMHC/)^40,41^. The corresponding protein accession identification numbers are: SARS-CoV-2 isolate, Wuhan-Hu-1 NCBI: YP_009724390.1, SARS-CoV-1 NCBI: NP_828852.2, MERS NCBI: YP_009047204.1, HCoV(HKU1) NCBI: YP_173238.1, HCoV(OC43) NCBI: YP_009555241.1, HCoV(NL63) NCBI: YP_003767.1, HCoV(229E) NCBI: NP_073551.1. NFKDQVILL in the nucleocapsid phosphoprotein has been previously reported as a SARS-CoV-2 dominant T cell epitope^9^.

### In-vitro expansion of SARS-CoV-2-specific T cells

For the peptide screening test, PBMCs (3–5 × 10^6^ cells) were stimulated with the indicated peptide (each peptide 10 µg/mL) (summarized in Supplementary Table 3) in culture medium in the presence of IL-2 (100 U/mL) (Shionogi CO., LTD) and restimulated with 30Gy-irradiated autologous PBMCs pulsed with each peptide weekly. Then, these cells were analyzed on day 21. For determining CD8^+^ T cell response against Pep#3(QYI), CD8^+^ T cells (0.5–1 × 10^6^ cells) were isolated from PBMCs using CD8 MACS beads (Miltenyi Biotec), and the isolated cells were stimulated with irradiated Pep#3(QYI)-pulsed autologous PBMCs (1:1 ratio) in the presence of IL-2 (100 U/mL) and further restimulated with irradiated autologous PBMCs pulsed with Pep#3(QYI) on day 7. Then, the cells were analyzed at day 14. For determining CD8^+^ T cell response using 15mer peptides, PBMCs (3–5 × 10^6^ cells) were stimulated with the 15mer peptide mixture (each peptide 10 µg/mL) in culture medium in the presence of IL-2 (100 U/mL) and restimulated with irradiated autologous PBMCs pulsed with peptide mixture on day 7. Then, these cells were analyzed at day 14. Cultures were supplemented with R10 media (RPMI-1640 medium supplemented with 10% FBS, 55 mM 2-ME, and 1% penicillin/streptomycin) containing IL-2 twice or thrice weekly. The harvested cells were washed out with R10, restimulated with or without the indicated peptide (10 µg/mL) in the presence of brefeldin A (50 μg/mL) (SIGMA) and monensin (750 ng/mL) (SIGMA)for 16 h, and analyzed using flow cytometry. To determine the threshold, we prepared PBMCs or sorted CD8T cells from UHDs and cultured them without peptide in the presence of IL-2. Two weeks later, the cells were restimulated with Pep#3 (control cells were not restimulated), and IFN-γ production was analyzed by intracellular cytokine staining. Then, we calculated the fold change of the % of IFN-γ^+^ CD8^+^ T cells in the restimulated group compared to that in the control group. Since the fold change in these experiments did not exceed 1.5 (mean ± 2SD: 0.94 ± 0.4) (Supplementary Fig. 1c), we set the threshold at 1.5.

### Flow cytometry

Antibodies were purchased from BD Bioscience, Biolegend, or e-Bioscience (summarized in Supplementary Table 4). Following stimulation, the cells were incubated with Human TruStain FcX (BioLegend) in FACS buffer (PBS with 2% heat-inactivated FBS) for Fc-Blockade and then stained with surface antibodies, CD3-PE/Cy7, CD4-PerCP/Cy5, and CD8-BUV737 or FITC and Live/Dead Fixable Aqua or Violet Stain (Life Technologies) for 30 min. For intracellular cytokine staining, the cells were washed twice with 2%FBS in PBS, fixed and permeabilized using BD Cytofix/Cytoperm solution and, then, intracellularly stained with anti-IFN-γ-APC, IL-2-BUV786, TNF-α-PE or IL-10-PE for further 30 min. For degranulation assay, we added anti-CD107a-Alexa488 in the culture during restimulation for 6 h. Then, after Fc-Blocking, the cells were stained with surface antibodies, CD3-PE/Cy7, CD4-PerCP/Cy5 and CD8-BUV737 and Live/Dead Fixable Aqua or Violet Stain (Life Technologies) for 30 min and fixed and permeabilized using BD Cytofix/Cytoperm solution and then intracellularly stained with anti-IFN-γ-APC for further 30 min. The LSR Fortessa X-20 instrument and FACSDiva (v 8.0.1) (BD Biosciences) or FlowJo software (v10.3B2) were used for data analysis.

### Analysis of cross-reactivity

The established Pep#3(QYI)-specific CD8^+^ T cell lines were restimulated with Pep#3(QYI) or the relevant peptide of HCoV (each peptide 10 μg/mL) and analyzed for cytokine production via intracellular cytokine staining. We used a graded dose of the peptide, ranging from 0.1 μg/mL to 10 μg/mL, to assess TCR avidity for each stimulating peptide. TCR avidity was assessed by %IFN-γ in CD8^+^ T cells. Furthermore, EC_50_ was estimated by calculating the peptide concentration required to reach one-half maximum %IFN-γ reached in our assay.

### Single-cell TCR sequencing and construction of retroviral TCR vector

Single-cell TCR sequencing was performed as below. All primers used are listed in Supplementary Table 5. CoV-2 pep#3-specific CD8^+^ T cell lines were restimulated with anti-human CD107a-BV421 in the presence or absence of 10 µM CoV-2 pep#3 for 6 h and then stained with anti-human CD8-PE and Aqua. The peptide-specific CD8^+^CD107a^+^ cells were sorted as single cells into 5 μL of RT-PCR mix in a 96-well plate, using FACS Aria III (BD Biosciences). The RT-PCR mix comprised 1 µL of 5× Prime STAR GXL Buffer, 0.45 µL RT-PCR primer mix, 0.4 µL of 2.5 mM dNTP, 0.05 µL of 40 U/µL RNase Inhibitor, 200 U/µL PrimeScript II Reverse Transcriptase (Takara Bio Inc,), 0.05 µL of 1.25 U/µL PrimeSTAR GXL DNA Polymerase (Takara Bio Inc.), and 2.95 µL nuclease-free water. The program used for the one-step RT-PCR was as follows: 45 °C for 40 min, 98 °C for 1 min, and 35 cycles of 98 °C for 10 s, 55 °C for 15 s, and 68 °C for 1 min. The resultant PCR products were diluted 10-fold with nuclease-free water and used as templates for the 2nd-PCR. The 2nd PCR was performed using PrimeSTAR GXL with the pMXs-BamHI-InFusion primer and CA-rev2 primer for TCRα or CB-rev2 primer for TCRβ in a 10 µL reaction volume. The program for the 2nd-PCR was as follows: 98 °C for 1 min and 44 cycles of 98 °C for 10 s, 55 °C for 15 s, and 68 °C for 1 min. The 2nd-PCR products were treated with ExoSAP-IT PCR Product Cleanup Reagent (Thermo Fisher Scientific K.K.) and sequenced using the CA-rev3 primer for TCRα or CB-rev3 primer for TCRβ. Sequencing results were analyzed using the V-QUEST tool of the IMGT database (http://www.imgt.org/IMGT_vquest/input)^42^. For cloning TCR, VDJ and C regions were amplified from the 2nd-PCR products and PBMC-derived cDNAs of the donors, respectively. PrimeSTAR Max was used for amplification with the pMXs-BamHI-InFusion and CA-rev3 or CB-rev3 primer. Each fragment was cloned into BamHI-digested linearized pMXs-IRES-GFP for TCRα or -TdTomato for TCRβ by the InFusion HD reaction.

### Establishment of SKW-3-CD8AB-expressing cells

SKW-3 cells were maintained in R10 media. hCD8A-IRES-Puro and hCD8B-IRES-Puro VSV-G retroviruses were transduced into SKW-3 cells in the presence of 5 μg/mL polybrene (Millipore) via centrifugation at 2300 rpm for 90 min at 35 °C. CD8AB-expressing cells were stained with PE-CD8A (RPA-T8) and BV421-CD8B (2ST8.5H7) and sorted twice using FACS Aria III. SKW-3-CD8AB-expressing cells were maintained with 3 µg/mL puromycin (InvivoGen).

### TCR clonotyping and αβ TCR cell line generation

The concentrated TCR Vα and TCR Vβ viruses were used to infect 2 × 10^6^ SKW-3-CD8AB cells with 5 µg/mL polybrene (Millipore) via centrifugation for 1 h at 1500 × *g* at 35 °C. The virus was removed, and the medium for cell culture was replaced with fresh medium. TCR-transduced SKW-3-CD8AB cells were enriched by sorting the GFP^+^ tdTOMATO^+^ cells.

### Epitope identification and avidity analysis of TCR-transduced SKW-3-CD8AB cells

The transduced SKW-3-CD8AB cells were co-cultured with A24/CIR pulsed with the indicated peptide in a 1:1 ratio for 16 h. CD69 was measured to detect early T cell activation by flow cytometry. Graded titrations of the peptide (from 10 μg/mL to 10 fg/mL) were used to assess TCR avidity. TCR avidity was assessed via CD69 MFI. EC_50_ was estimated by calculating the peptide concentration required to reach one-half maximum of CD69 MFI reached in our assay.

### Cytotoxicity assays

CD8^+^ T-cell lines were cultured for three weeks and then harvested as effector cells. T-cell cytotoxicity assays were performed using the following methodology: CFSE (Molecular Probe)-labeled A24-CIR cells were pulsed with the indicated peptide or DMSO for 2 h and washed twice. Effector cells were cultured with 1 × 10^4^ target cells, at effector/target cell ratios of 12.5, 25, and 50, for 6 h and then stained with TO-PRO3 (Molecular Probe) immediately prior to their analysis to identify dead cells. Spontaneous target cell death (SD) was determined by labeling the target cells that were cultured alone. As a positive control for total cytotoxicity (TD), the labeled target cells were permeabilized with BD Cytofix/Cytoperm reagent (BD Pharmingen). Specific lysis was calculated using the following formula: (Sample − SD/TD − SD) × 100. The cells were analyzed using flow cytometry.

### Protein expression and purification

HLA-A24 α chain and β-2-microglobulin with an N-terminal histidine tag and a TEV protease cleavage site were co-expressed in a cell-free expression system^43,44^. The cell-free reaction mixture was prepared with 0.15 mg/mL of each peptide (CoV2, 229E, and HKU1). The HLA-A*24:02•peptide complexes were purified via chromatography using a HisTrap column (Cytiva) and subjected to TEV protease digestion. Protein solutions were then applied to the HisTrap column to remove the histidine-tag. The flow-through fractions were subsequently purified using HiTrap Q and Superdex200 gel filtration chromatography (Cytiva). The HLA-A*24:02•peptide complexes were concentrated in 20 mM Tris-HCl buffer (pH 8.0) containing 50 mM NaCl.

### Crystallization and data collection

Crystallization was performed using the sitting-drop vapor-diffusion method at 293 K. The HLA-A*24:02•CoV2 peptide complex crystals were grown in 1.0 M Na/K phosphate (pH 8.2) at a concentration of 6.6 mg/mL. The HLA-A*24:02•229E peptide complex crystals were grown in 0.2 M sodium citrate and 20% PEG3350 at a concentration of 6.9 mg/mL. The HLA-A*24:02•HKU1 peptide complex crystals were grown in 0.1 M Tris-HCl (pH 8.5) and 25% PEG3350 at a concentration of 2.4 mg/mL. Data collection was carried out at 100 K with 10% glycerol as a cryoprotectant. Data were collected at BL26B2^45–48^ and BL32XU^49^ of the SPring-8 synchrotron beamlines with a wavelength of 1.000 Å. The diffraction data were processed and scaled using the ZOO/KAMO: Automated Data Processing System for Microcrystals^50,51^ and the CCP4 software suite^52^.

### Structure determination and refinement

The structures were determined using the molecular replacement method (MR) with Phaser^53^. The coordinates of the HLA-A*24:02 complex with the newly identified peptide (PDB ID: 4F7M) were used as the search model. The model was corrected and further refined using Phenix^54^ and Coot^55^ The Ramachandran plot indicated that 97.4%/2.6% for the CoV2 complex, 97.4%/2.6% for the 229E complex, and 97.7%, 2.3% for HKU1 complex are in favored and allowed regions. Data collection and refinement statistics are summarized in Table 1. Ribbon models in the figures are depicted using PyMOL software (http://www.pymol.org).

### Statistics and reproducibility

Statistical analysis was performed using StatMate V (Nihon 3B Scientific Inc.). Fisher’s/Chi-square tests were used to compare ratio difference between two groups. Data were analyzed using Mann–Whitney *U*-test for two independent groups and Turkey or Neuman–Keuls tests for non-parametric multiple comparisons. *p* < 0.05 was considered statistically significant. The number of biological replicates or sample size are given in figure legend.

### Reporting summary

Further information on research design is available in the Nature Research Reporting Summary linked to this article.

## Supplementary information

Supplementary Information

Description of Additional Supplementary Files

Supplementary Data 1

Reporting Summary

## Data Availability

The structures of HLA-A*24:02•peptide (CoV2, 229E, and HKU1) complexes were deposited in the RCSB Protein DataBank (PDB) under accession codes 7EJL, 7EJM, and 7EJN respectively. The TCR sequence data were deposited in the BioProject database (PRJNA779816). All source data underlying the graphs presented in the main figures are available in Supplementary Data 1.
