# Supplementary material for: Identification of TCR repertoires in functionally competent cytotoxic T cells cross-reactive to SARS-CoV-2
Source: Commun Biol. 2021 Dec 2;4:1365. doi: 10.1038/s42003-021-02885-6 (PMC8640030; doi:10.1038/s42003-021-02885-6)
Supplement: Supplementary file 1 — Supplementary Information [file 42003_2021_2885_MOESM1_ESM.pdf]

Supplementary Information

Identification of TCR repertoires in functionally competent cytotoxic T cells cross-reactive to SARS-CoV-2

Kanako Shimizu, Tomonori Iyoda, An Sanpei, Hiroshi Nakazato, Masahiro Okada, Shogo Ueda, Miyuki Kato-Murayama, Kazutaka Murayama, Mikako Shirouzu, Naoko Harada, Michihiro Hidaka, and Shin-ichiro Fujii

Inventory:

-Supplementary Figures

Supplementary Figure 1. Screening of immunodominant peptides in SARS-CoV-2 spike protein and their homology between SARS-CoV-2 and other human coronaviruses.

Supplementary Figure 2. IL-10 production by Pep#3(QYI)-specific CD8$^+$ T cell line.

Supplementary Figure 3. Homology of amino acid sequences

Supplementary Figure 4. 15mer peptide mix-responding CD8$^+$ T cells in HLA-A*24:02 positive patients with hematological malignancy (HM).

Supplementary Figure 5. TCR repertoire from Pep#3(QYI)-specific CD8 T cells.

-Supplementary Tables

Supplementary Table 1. Characteristics of HLA-A24$^+$ healthy volunteers

Supplementary Table 2. Characteristics of HLA-A24$^+$ patients with hematological malignancies

Supplementary Table 3. List of peptides

Supplementary Table 4. List of antibodies

Supplementary Table 5. Single cell TCR sequence and cloning primer list

Supplementary Figure 1

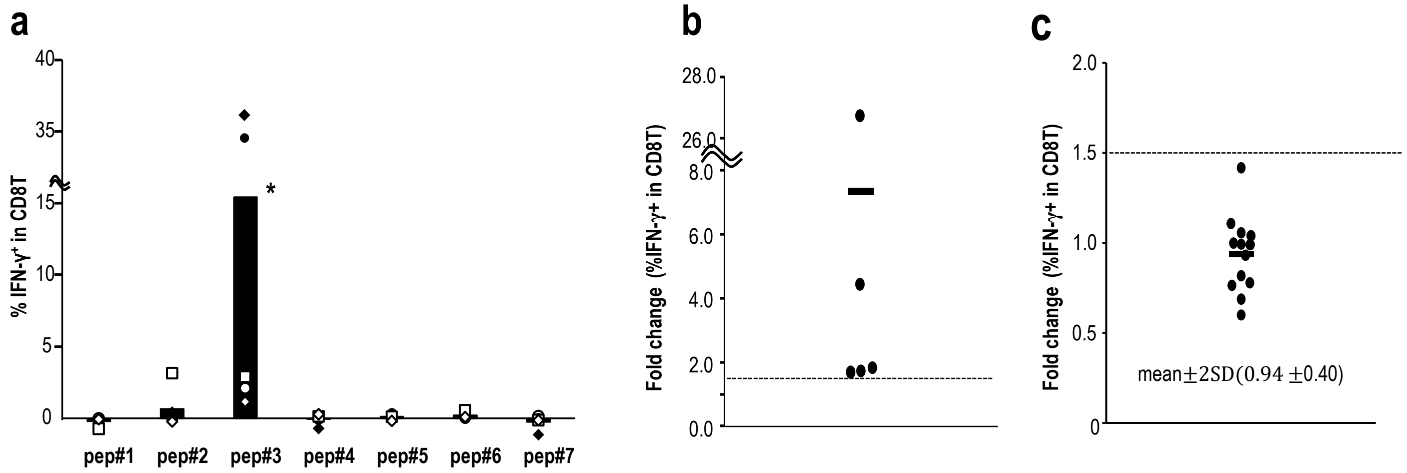

d

Pep#1 VYSTGSNVF

SARS-CoV-2-S	V------------YSTGS------------------NVF
SARS-CoV-1-S	I------------YSTGN------------------NVF
MERS-S	FGSVACEHISSTMSQYSRSTRSMLKRRDSTYGPL
HCoV-HKU1-S	YRNLKCSYVLNNIS-----LTTQ----PYF ----
HCoV-OC43-S	FRNIKCNYVFNN-S---------LTRQLQPINYF
HCoV-NL63-S	LQLPNFYYVSNGGN-------------------
HCoV-229E-S	VELPKFFYASNGTY-------------------

Pep#2 VYSSANNCTF

SARS-CoV-2-S	VYSSANNC-------------TF
SARS-CoV-1-S	IFDNAFNC-------------TF
MERS-S	EYFNLRNC-------------TF
HCoV-HKU1-S	FDKSEPLC---------------LF
HCoV-OC43-S	LDTGVVSC-------------LY
HCoV-NL63-S	LLTNGSTLVDGVSRLY
HCoV-229E-S	LLTNTSSVVDGVVRSF

Pep#4 NYNYLYRLF

SARS-CoV-2-S	NYNYLYRLF
SARS-CoV-1-S	NYNYKYRYL
MERS-S	PLKYSYINK
HCoV-HKU1-S	NYN------
HCoV-OC43-S	RFN------
HCoV-NL63-S	YLKSGTCPF
HCoV-229E-S	SINTGNCPF

Pep#5 YFPLQSYGF

SARS-CoV-2-S	YFPLQSYGF
SARS-CoV-1-S	YWPLNDYGF
MERS-S	------------------
HCoV-HKU1-S	RYGFNNFN-
HCoV-OC43-S	RFGFIEDSV
HCoV-NL63-S	NFPLEATWH
HCoV-229E-S	AMPIVANWA

Pep#6 TYVPAQEKNF

SARS-CoV-2-S	TYVPAQEKN
SARS-CoV-1-S	TYVPSQERN
MERS-S	GYYPSNHIE
HCoV-HKU1-S	SYKPISFKT
HCoV-OC43-S	SYVPTKYVT
HCoV-NL63-S	VLLPTDYKN
HCoV-229E-S	VLLPTQYKD

Pep#7 NFKDQVILL

SARS-CoV-2-N	NFKDQVILL
SARS-CoV-1-N	QFKDNVILL
MERS-N	NYNKWLELL
HCoV-HKU1-N	GFETIMKVL
HCoV-OC43-N	GFETIMKVL
HCoV-NL63-N	KDNKNLPKF
HCoV-229E-N	KDHPHLGKF

Supplementary Fig. 1 Screening of immunodominant peptides in SARS-CoV-2 spike protein and their homology between SARS-CoV-2 and other human coronaviruses.

a. As shown in Fig.1 **b**, the comparison of peptide-specific CD8$^+$ T cells was shown by the frequency of IFN-γ$^+$ CD8$^+$ T cell response to each peptide in the five UHDs. Data were calculated by subtracting the frequency of IFN-γ$^+$ CD8$^+$ T cells cultured without peptide restimulation (CD8T (pep-)) from that with peptide restimulation (CD8T (pep+)). The black bar and other symbols represent the average of the five UHDs and each data, respectively. $*p < 0.05$ (Tukey's test) (Pep#3(QYI) vs others). **b.** Fold change of Pep#3(QYI)-specific IFN-γ$^+$ CD8$^+$ T cells was calculated as restimulation(+)/restimulation (-). **c.** PBMCs or sorted CD8$^+$ T cells from 13 UHDs were cultured in the absence of peptide and restimulated with or without Pep#3 at day 14. Fold change of Pep#3(QYI)-specific IFN-γ$^+$ CD8$^+$ T cells was calculated as restimulation(+)/restimulation (-) (mean ± 2SD: 0.94±0.4) **d.** For each epitope, the respective section from a global sequence alignment between SARS-CoV-2 and other human coronaviruses (SARS-CoV-2, MERS, HKU-1, OC43, NL63 and 229E) is shown. Identical residues are color-coded from white (no identity) to violet (100% identity).

Supplementary Figure 2

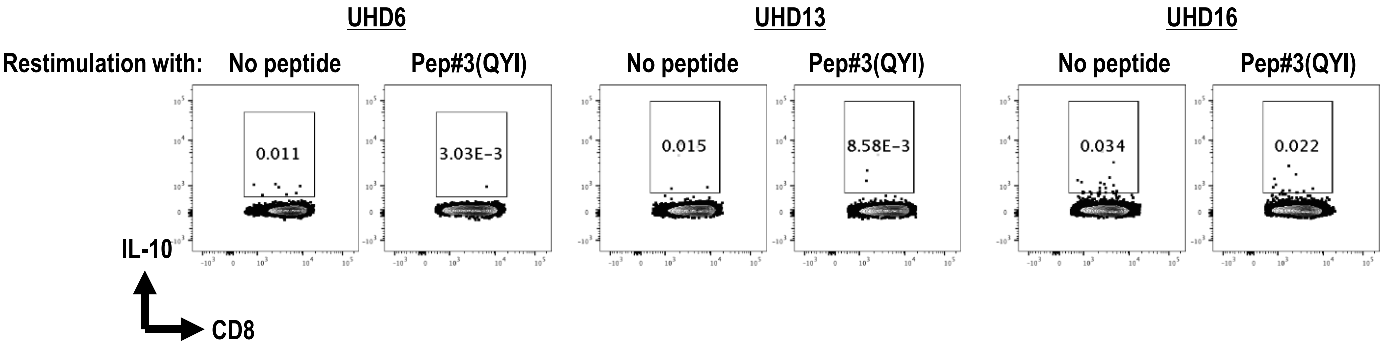

Supplementary Fig. 2 IL-10 production by Pep#3(QYI)-specific CD8^{+} T cell line.
As same shown in Fig 1e, Pep#3(QYI)-specific CD8^{+} T cell lines were restimulated with or without Pep#3(QYI), and IL-10 production was measured via ICS analysis. Flow cytometry data are from representative donors.

Supplementary Figure 3

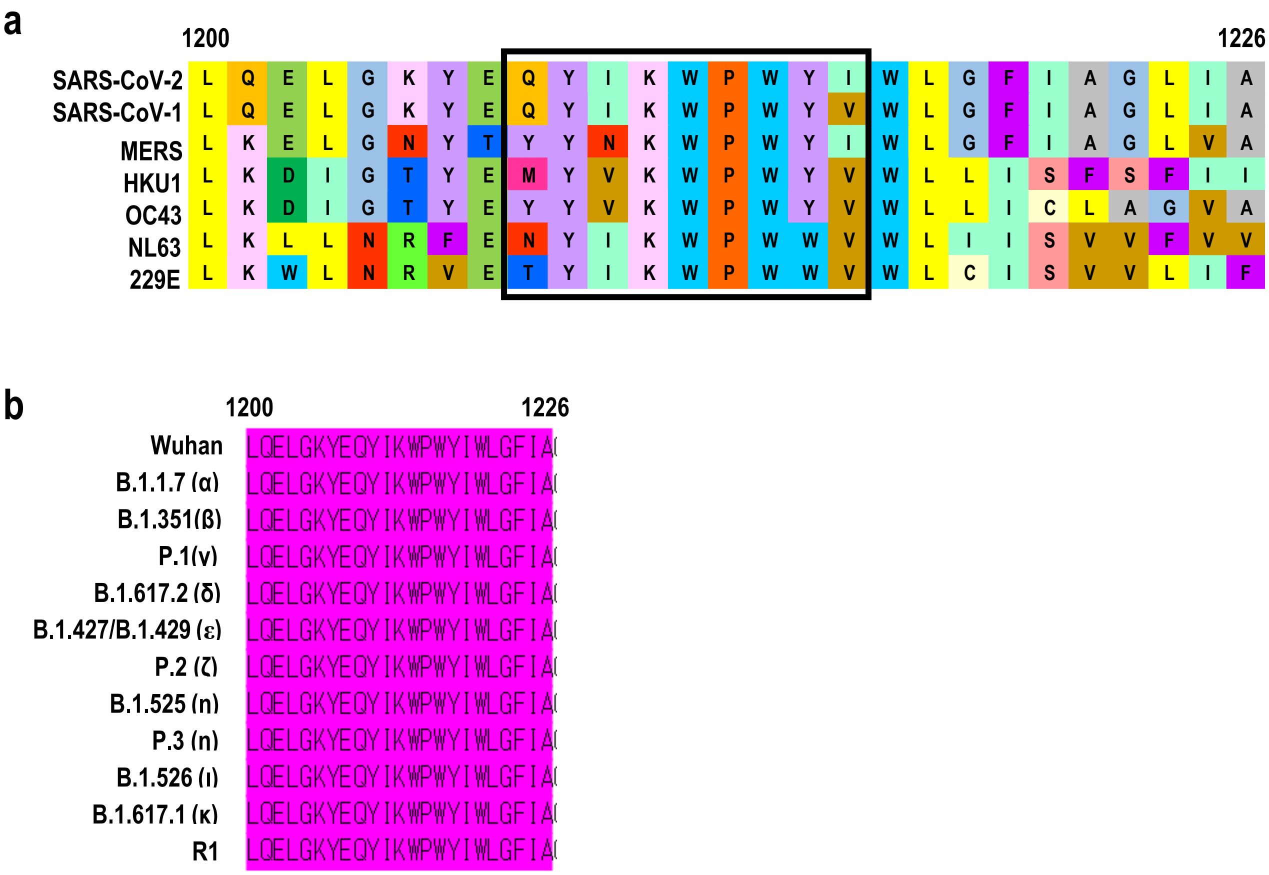

Supplementary Fig. 3 Homology of amino acid sequences
a. Homology of amino acid sequences in SARS-CoV-2 Spike protein$_{1200\text{-}1226}$ compared to that in the relevant region of other human coronaviruses. Identical residues are same color-coded. **b.** Homology of amino acid sequences of SARS-CoV-2 Spike protein$_{1200\text{-}1226}$ of Wuhan strain compared to that of other variants. Identical residues are color-coded to violet (100% identity).

Supplementary Figure 4

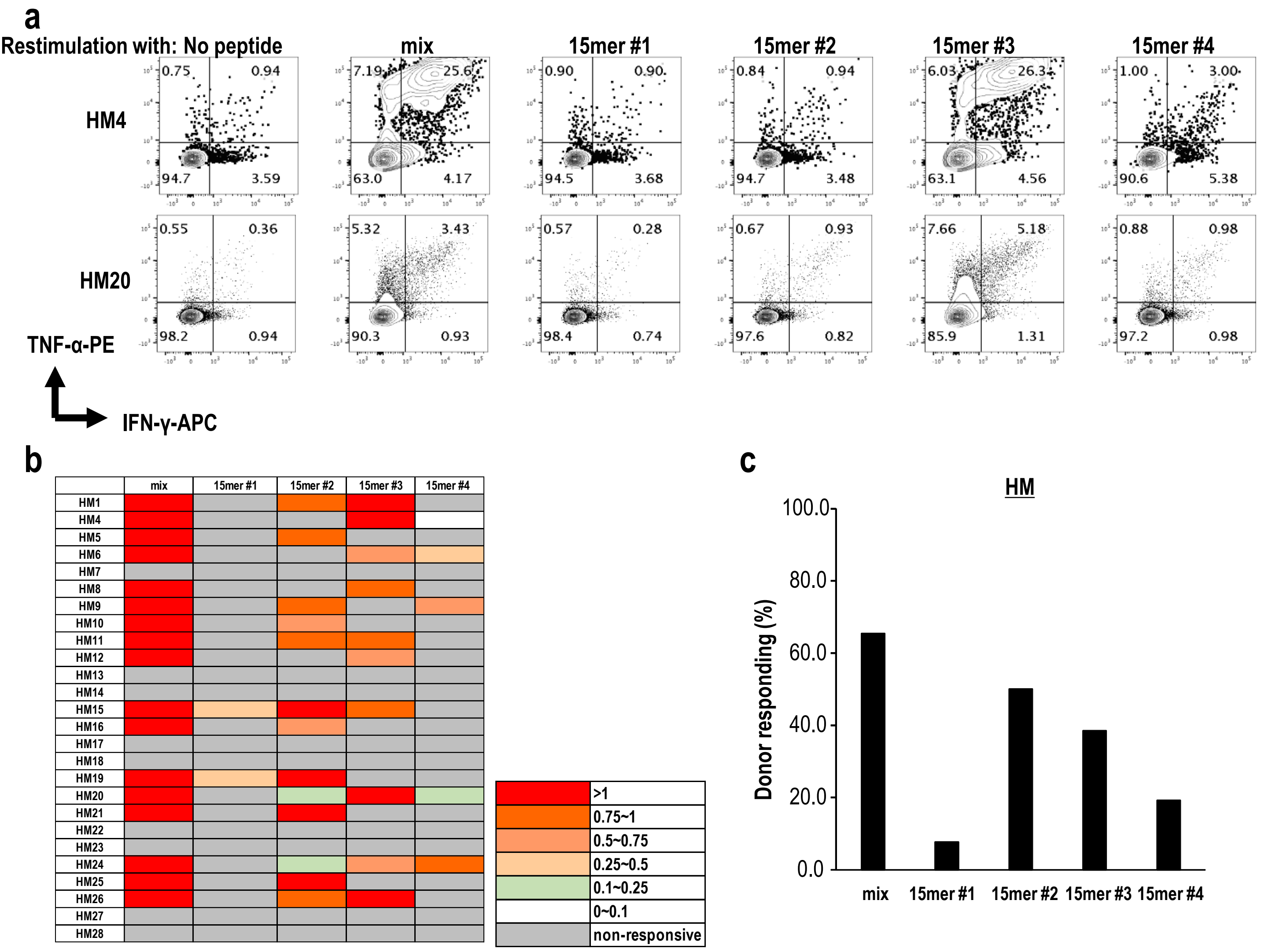

Supplementary Fig. 4 15mer peptide mix-responding CD8⁺ T cells in HLA-A*24:02 positive patients with hematological malignancy (HM).

As shown in **Fig. 3e**, PBMCs from patients with HM were cultured in the presence of 15mer peptide mix. Two weeks later, the cultured cells were restimulated with or without the peptide mix or each 15mer peptide for 16 h in the presence of monensin and brefeldin. The frequency of IFN-γ^+TNF-α^+ $CD8^+$ T cells as responding $CD8^+$ T cells was measured after gating the $CD8^+$ T cells, using ICS analysis. **a.** Flow cytometry data from representative donors (HM4 and HM20). **b.** Summary of the 15-mer peptide-specific $CD8^+$ T cells. The peptide-specific $CD8^+$ T cell frequency was calculated by subtracting the frequency of $CD8^+$ T (pep-) from that of $CD8^+$ T(pep+). Data indicate the ratio of the frequency of each 15mer peptide-responding $CD8^+$ T cells to that of 15mer peptide mix responding $CD8^+$ T cells. (n=26) **c.** Percentage of responding donors who had $CD8^+$ T cells specific (against) each 15-mer peptide.

Supplementary Figure 5

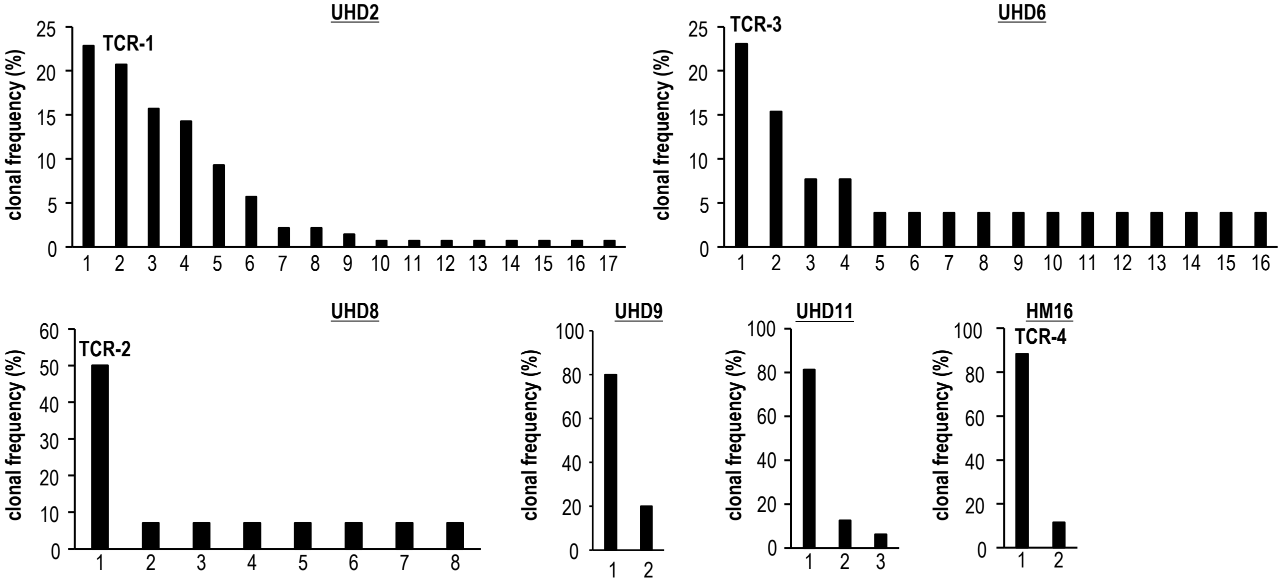

Supplementary Fig. 5 TCR repertoire from Pep#3(QYI)-specific CD8 T cells
As described in Fig. 7, the single-cell TCR sequences in Pep#3(QYI)-specific $CD8^+$ T cells were analyzed from 227 $CD107a^+CD8^+$ T cells from five UHD and one HM-patient. Forty-four clones were detected from 227 $CD8^+$ T cells. Data represent the clonal frequency of each TCR clonotype in the individuals.

Supplementary Table 1. Characteristics of HLA-A24^{+} healthy volunteers

Sampling time	HLA-A24+ healthy Donor	Sex	Age	anti-SARS-CoV-2 IgG	COVID-19 Human IgM IgG Rapid test
2020	UHD1	Male	57	negative	negative
	UHD2	Male	34	negative	negative
	UHD3	Male	39	negative	negative
	UHD4	Male	45	negative	negative
	UHD5	Male	35	negative	negative
	UHD6	Male	50	negative	negative
	UHD7	Male	50	negative	negative
	UHD8	Female	37	negative	negative
	UHD9	Female	20	negative	negative
	UHD10	Female	42	N.D.	negative
2004-2010	UHD11	no information but from Red cross		N.D.	N.D.
	UHD12	no information but from Red cross			
	UHD13	no information but from Red cross			
	UHD14	no information but from Red cross			
	UHD15	no information but from Red cross			
	UHD16	no information but from Red cross			
	UHD17	no information but from Red cross			
	UHD18	no information but from Red cross			
	UHD19	no information but from Red cross			
	UHD20	no information but from Red cross			
	UHD21	no information but from Red cross			

N.D. not done

Supplementary Table 2. Characteristics of HLA-A24+ patients with hematological malignancies

Patient ID	F/M	Age	Disease	Status	Treatment within 1 year	Treatment over a year ago	WBC(x10²/ μL)	Hb (g/dL)	Plts (x10⁴/μL)	anti-SARS CoV-2 IgG	COVID-19 Human IgM IgG Rapid test
HM-1	M	50	AML NOS	non CR	IDA AraC induction		25.7	14	16.5	negative	negative
HM-2	M	78	MM IgG	CR	Bd		77	12	16.4	negative	negative
HM-3	F	48	MM IgG	PR	Rd		28.4	12.5	16.8	negative	negative
HM-4	M	53	AML (inv16)	CR	IDA AraC induction		53.2	14.2	15.9	negative	negative
HM-5	M	73	MM IgG	PR	Rd		38.7	10.3	11.8	negative	negative
HM-6	M	76	MM IgA	non CR	IRd		69.8	14.1	12.6	negative	negative
HM-7	F	78	AML NOS	CR	CAG		66.4	7.6	3.9	negative	negative
HM-8	F	84	CML	DMR		Imatinib	50.8	12.4	26.9	negative	negative
HM-9	F	63	MM IgG	VGPR	Rd	high dose L-PAM+ autologous PBSCT	43.5	13.3	20.9	negative	negative
HM-10	F	90	MM IgA	non CR	MP		15	10.2	16.2	negative	negative
HM-11	M	66	AML t(8;21)	CR	IDA AraC induction, HD-AraC		59.2	10.5	11.3	negative	negative
HM-12	F	74	MM IgA	non CR	Ird	Rd , Bd	33	9.1	16.9	negative	negative
HM-13	F	68	MM IgG	CR	Rd	high dose L-PAM+ autologous PBSCT	33.8	10.7	24.8	negative	negative
HM-14	F	37	AML M3	CR	Tamibarotene	IDA/AraC, MIT/AraC, DNR/AraC, IDA/AraC	43.9	13.2	22.7	negative	negative
HM-15	M	83	AML	CR	CAG		56	14.5	12.7	negative	negative
HM-16	F	50	AML (inv16)	CR		IDA/AraC, HD-AraC x3	55.4	10	32	negative	negative
HM-17	M	71	MM IgG	VGPR	Bd		83.8	13.7	16.3	negative	negative
HM-18	M	69	MM IgA	non CR	Pd	BD, Rd, ERd, DRd, IRd	28.6	7.2	17.8	negative	negative
HM-19	M	64	MM IgG	VGPR	DBd	high dose L-PAM+ autologous PBSCT	34.1	14.5	13.7	negative	negative
HM-20	M	41	AML M3	CR	DNR/AraC, ATRA		39.8	9.2	27.1	negative	negative
HM-21	M	59	DLBCL	CR		R-CHOP x6	57.9	15.8	18.9	negative	negative
HM-22	F	67	MM IgG	non CR	IRd	Rd, BD, Pd, ERd, DRd, Kd	21.89	9.4	13.9	negative	negative
HM-23	F	79	CML	DMR	Imatinib		42.3	10.6	22.6	negative	negative
HM-24	M	44	AML M3	CR	Tamibarotene	IDA/AraC, MIT/AraC, DNR/AraC, IDA/AraC	56.5	14.4	22.1	negative	negative
HM-25	F	78	CML	MMR	Imatinib	Dasatinib	56.5	10.8	21.1	negative	negative
HM-26	M	85	AML	CR		CAG	63.1	14.3	16.1	negative	negative
HM-27	M	61	AML t(8;21)	CR		IDA/AraC, MIT/AraC, DNR/AraC	43.1	13.3	11.2	negative	negative
HM-28	F	76	CML	DMR		Dasatinib	54.5	13.7	26.7	negative	negative

AML; Acute myelogenous leukemia, MM; Multiple myeloma, CML; Chronic myelogenous leukemia, DLBCL; Diffuse large B cell lymphoma, NOS; not otherwise specified
CR: complete response, PR; partial response, DMR; deep molecular response, VGPR; very good PR, MMR; major molecular response
Bd (bortezomib +dexamethasone), Rd (lenalidomide+dexamethasone), Ird (izazomib +lenalidomide+dexamethasone), Pd (pomalidomide+dexamethasone), DBd (daratumumab+bortezomib +dexamethasone)
CAG; cytarabine+aclarubicin+G-CSF, PBSCT; peripheral blood stem cell transplantation

Supplementary Table 3. List of peptides

Peptide size	Peptide sequence	Peptide name
9-mer peptides	NFKDQVILL	Nucleocapside protein 345-353
	VYSTGSNVF	Spike protein 635-643
	VYSSANNCTF	Spike protein 159-168
	QYIKWPWYI	Spike protein 1208-1216 Pep#3(QYI)
	NYNYLYRLF	Spike protein 448-456
	YFPLQSYGF	Spike protein 489-497
	TYVPAQEKNF	Spike protein 1066-1075
	QYIKWPWYV	SARS-CoV-1
	YYNKWPWYI	MERS
	MYVKWPWYV	HKU1
	YYVKWPWYV	OC43
	NYIKWPWWV	NL63
	TYIKWPWWV	229E
15-mer library	LQELGKYEQYIKWPW	15 mer #1
	GKYEQYIKWPWYIWL	15 mer #2
	QYIKWPWYIWLGFIA	15 mer #3
	WPWYIWLGFIAGLIA	15 mer #4
9-mer library	LQELGKYEQ	S2-pep #1200
	QELGKYEQY	S2-pep #1201
	ELGKYEQYI	S2-pe #p1202
	LGKYEQYIK	S2-pep #1203
	GKYEQYIKW	S2-pep #1204
	KYEQYIKWP	S2-pep #1205
	YEQYIKWPW	S2-pep #1206
	EQYIKWPWY	S2-pep #1207
	QYIKWPWYI	S2-pep #1208
	YIKWPWYIW	S2-pep #1209
	IKWPWYIWL	S2-pep #1210
	KWPWYIWLG	S2-pep #1211
	WPWYIWLGF	S2-pep #1212
	PWYIWLGFI	S2-pep #1213
	WYIWLGFIA	S2-pep #1214
	YIWLGFIAG	S2-pep #1215
	IWLGFIAGL	S2-pep #1216
	WLGFIAGLI	S2-pep #1217
	LGFIAGLIA	S2-pep #1218

Supplementary Table 4. List of antibodies

Antibodies	Source	Dilution	Identifier
Mouse anti-human CD3 (UCHT1) PE/Cyanine7	Biolegend	1:500	Cat#300420; RRID:AB_439781
Mouse anti-human CD4 (OKT4) PerCP/Cy5.5	Biolegend	1:100	Cat#317428; RRID:AB_1186122
Mouse anti-human CD4 (RPA-T4) PerCP/Cyanine5.5	Biolegend	1:100	Cat#300530; RRID:AB_893322
Mouse anti-human CD8a (RPA-T8) FITC	Biolegend	1:200	Cat#301006; RRID:AB_314124
Mouse Anti-Human CD8 (SK1) BUV737	BD	1:500	Cat#564629
Mouse anti-human CD69 (FN50) APC	Biolegend	1:400	Cat#310904; RRID:AB_314839
Mouse anti-human CD107a (H4A3) BV421	Biolegend	1:100	Cat#328626; RRID:AB_11203537
Mouse anti-human CD107a (H4A3) Alexa Fluor 488	Biolegend	1:40	Cat#328610; RRID:AB_1227504
Rat anti-human IL-2 (MQ1-17H12) BV785	Biolegend	1:100	559337; RRID:AB_2566471
Rat Anti-Human and Viral IL-10 (JES3-9D7) PE	BD	1:20	Cat#559337
Mouse anti-human TNF-α (MAb11) PE	Biolegend	1:200	Cat#502909; RRID:AB_315261
Mouse anti-human IFN-γ (B27) APC	Biolegend	1:100	Cat#506510; RRID:AB_315443
Mouse Anti-Human HLA-A24 (17A10) FITC	MBL	1:100	Cat#K0208-4; RRID:AB_592231

Supplementary Table 5. Single cell TCR sequence and cloning primer list

Primer Name	Primer Sequence	PCR reaction
TRAC-inf-for	GTCTGCCTATTCACCGATTTTG	TRAC InFusion
TRAC-inf-revcom	CACCACACTGG GATC c TCAGCTGGACCACAGCCGCAG	TRAC InFusion
AV1-1	AGTTAATTAAG GATC c gcc atgtggggagctttccttct	RT-PCR 0.44 µM
AV2	AGTTAATTAAG GATC c gcc atggctttgcagagcactct	RT-PCR 0.44 µM
AV3	AGTTAATTAAG GATC c gcc atggcctctgcacccatctc	RT-PCR 0.44 µM
AV4	AGTTAATTAAG GATC c gcc atgaggcaagtggcgagagt	RT-PCR 0.44 µM
AV5	AGTTAATTAAG GATC c gcc atgaagacatttgctggatt	RT-PCR 0.44 µM
AV6	AGTTAATTAAG GATC c gcc atggagtcattcctgggagg	RT-PCR 0.44 µM
AV7	AGTTAATTAAG GATC c gcc atggagaagatgcggagacc	RT-PCR 0.44 µM
AV8-1	AGTTAATTAAG GATC c gcc atgctcctgttgctcatacc	RT-PCR 0.44 µM
AV8-2	AGTTAATTAAG GATC c gcc atgctcctgctgctcgtccc	RT-PCR 0.44 µM
AV8-3	AGTTAATTAAG GATC c gcc atgctcctggagcttatccc	RT-PCR 0.44 µM
AV9-1	AGTTAATTAAG GATC c gcc atgaattcttctccaggacc	RT-PCR 0.44 µM
AV9-2	AGTTAATTAAG GATC c gcc atgaactattctccaggctt	RT-PCR 0.44 µM
AV10	AGTTAATTAAG GATC c gcc atgaaaaagcatctgacgac	RT-PCR 0.44 µM
AV12-1	AGTTAATTAAG GATC c gcc atgatatccttgagagtttt	RT-PCR 0.44 µM
AV12-3	AGTTAATTAAG GATC c gcc atgatgaaatccttgagagt	RT-PCR 0.44 µM
AV13-1	AGTTAATTAAG GATC c gcc atgacatccattcgagctgt	RT-PCR 0.44 µM
AV13-2	AGTTAATTAAG GATC c gcc atggcaggcattcgagcttt	RT-PCR 0.44 µM
AV14-DV4	AGTTAATTAAG GATC c gcc atgtcactttctagcctgct	RT-PCR 0.44 µM
AV16	AGTTAATTAAG GATC c gcc atgaagcccaccctcatctc	RT-PCR 0.44 µM
AV17	AGTTAATTAAG GATC c gcc atggaaactctcctgggagt	RT-PCR 0.44 µM
AV18	AGTTAATTAAG GATC c gcc atgctgtctgcttcctgctc	RT-PCR 0.44 µM
AV19	AGTTAATTAAG GATC c gcc atgctgactgccagcctgtt	RT-PCR 0.44 µM
AV20	AGTTAATTAAG GATC c gcc atggagaaaatgttggagtg	RT-PCR 0.44 µM
AV21	AGTTAATTAAG GATC c gcc atggagaccctcttgggcct	RT-PCR 0.44 µM
AV22	AGTTAATTAAG GATC c gcc atgaagaggatattgggagc	RT-PCR 0.44 µM
AV23-DV6	AGTTAATTAAG GATC c gcc atggacaagatcttaggagc	RT-PCR 0.44 µM
AV24	AGTTAATTAAG GATC c gcc atggagaagaatcctttggc	RT-PCR 0.44 µM
AV25	AGTTAATTAAG GATC c gcc atgctactcatcacatcaat	RT-PCR 0.44 µM
AV26-1	AGTTAATTAAG GATC c gcc atgaggctggtggcaagagt	RT-PCR 0.44 µM
AV26-2	AGTTAATTAAG GATC c gcc atgaagttggtgacaagcat	RT-PCR 0.44 µM
AV27	AGTTAATTAAG GATC c gcc atggtcctgaaattctccgt	RT-PCR 0.44 µM
AV29-DV5	AGTTAATTAAG GATC c gcc atggccatgctcctgggggc	RT-PCR 0.44 µM
AV30	AGTTAATTAAG GATC c gcc atggagactctcctgaaagt	RT-PCR 0.44 µM
AV34	AGTTAATTAAG GATC c gcc atggagactgttctgcaagt	RT-PCR 0.44 µM
AV35	AGTTAATTAAG GATC c gcc atgctccttgaacatttatt	RT-PCR 0.44 µM
AV36-DV7	AGTTAATTAAG GATC c gcc atgatgaagtgtccacaggc	RT-PCR 0.44 µM
AV38-1	AGTTAATTAAG GATC c gcc atgacacgagttagcttgct	RT-PCR 0.44 µM
AV38-2-DV8	AGTTAATTAAG GATC c gcc atggcatgccctggcttcct	RT-PCR 0.44 µM
AV39	AGTTAATTAAG GATC c gcc atgaagaagctactagcaat	RT-PCR 0.44 µM
AV40	AGTTAATTAAG GATC c gcc atgaactcctctctggactt	RT-PCR 0.44 µM
AV41	AGTTAATTAAG GATC c gcc atggtgaagatccggcaatt	RT-PCR 0.44 µM
CA-rev1	AGGTTCGTATCTGTTTCAAAGCTT	RT-PCR 2.22 µM
pMXs-BamHI-InFusion	AGTTAATTAAG GATC c gcc	2nd/3rd PCR
CA-rev2	TGTGACACATTTGTTTGAGAA	2nd PCR

CA-rev3	GGTGAATAGGCAGACAGACTT	Sequence/3rd PCR
TRBC-inf-for	GGTGTGCCTGGCCACAGGCTTC	TRBC1/2 InFusion
TRBC1-inf-revcom	CACCACACTGG GATC c TCAGAAATCCTTTCTCTTGAC	TRBC1 InFusion
TRBC2-inf-revcom	CACCACACTGG GATC c CTAGCCTCTGGAATCCTTTC	TRBC2 InFusion
BV2	AGTTAATTAAG GATC c gcc atggatacctggctcgtatg	RT-PCR 0.44 µM
BV3-1	AGTTAATTAAG GATC c gcc atgggctgcaggctcctctg	RT-PCR 0.44 µM
BV4-1	AGTTAATTAAG GATC c gcc atgggctgcaggctgctctg	RT-PCR 0.44 µM
BV5-1	AGTTAATTAAG GATC c gcc atgggctccaggctgctctg	RT-PCR 0.44 µM
BV5-4	AGTTAATTAAG GATC c gcc atgggccctgggctcctctg	RT-PCR 0.44 µM
BV5-6	AGTTAATTAAG GATC c gcc atgggccccgggctcctctg	RT-PCR 0.44 µM
BV5-8	AGTTAATTAAG GATC c gcc atgggacccaggctcctctt	RT-PCR 0.44 µM
BV6-1	AGTTAATTAAG GATC c gcc atgagcatcgggctcctgtg	RT-PCR 0.44 µM
BV6-2	AGTTAATTAAG GATC c gcc atgagcctcgggctcctgtg	RT-PCR 0.44 µM
BV6-4	AGTTAATTAAG GATC c gcc atgagaatcaggctcctgtg	RT-PCR 0.44 µM
BV6-5	AGTTAATTAAG GATC c gcc atgagcatcggcctcctgtg	RT-PCR 0.44 µM
BV6-6	AGTTAATTAAG GATC c gcc atgagcatcagcctcctgtg	RT-PCR 0.44 µM
BV7-2	AGTTAATTAAG GATC c gcc atgggcaccaggctcctctt	RT-PCR 0.44 µM
BV7-3	AGTTAATTAAG GATC c gcc atgggcaccaggctcctctg	RT-PCR 0.44 µM
BV7-6	AGTTAATTAAG GATC c gcc atgggcaccagtctcctatg	RT-PCR 0.44 µM
BV7-7	AGTTAATTAAG GATC c gcc atgggtaccagtctcctatg	RT-PCR 0.44 µM
BV7-9	AGTTAATTAAG GATC c gcc atgggcaccagcctcctctg	RT-PCR 0.44 µM
BV9	AGTTAATTAAG GATC c gcc atgggcttcaggctcctctg	RT-PCR 0.44 µM
BV10-1	AGTTAATTAAG GATC c gcc atgggcacgaggctcttctt	RT-PCR 0.44 µM
BV10-2	AGTTAATTAAG GATC c gcc atgggcaccaggctcttctt	RT-PCR 0.44 µM
BV10-3	AGTTAATTAAG GATC c gcc atgggcacaaggttgttctt	RT-PCR 0.44 µM
BV11-1	AGTTAATTAAG GATC c gcc atgagcaccaggcttctctg	RT-PCR 0.44 µM
BV11-3	AGTTAATTAAG GATC c gcc atgggtaccaggctcctctg	RT-PCR 0.44 µM
BV12-3	AGTTAATTAAG GATC c gcc atggactcctggaccttctg	RT-PCR 0.44 µM
BV12-4	AGTTAATTAAG GATC c gcc atgggctcctggaccctctg	RT-PCR 0.44 µM
BV12-5	AGTTAATTAAG GATC c gcc atggccaccaggctcctctg	RT-PCR 0.44 µM
BV13	AGTTAATTAAG GATC c gcc atgcttagtcctgacctgcc	RT-PCR 0.44 µM
BV14	AGTTAATTAAG GATC c gcc atggtttccaggcttctcag	RT-PCR 0.44 µM
BV15	AGTTAATTAAG GATC c gcc atgggtcctgggcttctcca	RT-PCR 0.44 µM
BV16	AGTTAATTAAG GATC c gcc atgagcccaatattcacctg	RT-PCR 0.44 µM
BV18	AGTTAATTAAG GATC c gcc atggacaccagagtactctg	RT-PCR 0.44 µM
BV19	AGTTAATTAAG GATC c gcc atgagcaaccaggtgctctg	RT-PCR 0.44 µM
BV20-1	AGTTAATTAAG GATC c gcc atgctgctgcttctgctgct	RT-PCR 0.44 µM
BV24-1	AGTTAATTAAG GATC c gcc atggcctccctgctcttctt	RT-PCR 0.44 µM
BV25-1	AGTTAATTAAG GATC c gcc atgactatcaggctcctctg	RT-PCR 0.44 µM
BV27	AGTTAATTAAG GATC c gcc atgggcccccagctccttgg	RT-PCR 0.44 µM
BV28	AGTTAATTAAG GATC c gcc atgggaatcaggctcctgtg	RT-PCR 0.44 µM
BV29-1	AGTTAATTAAG GATC c gcc atgctgagtcttctgctcct	RT-PCR 0.44 µM
BV30	AGTTAATTAAG GATC c gcc atgctctgctctctccttgc	RT-PCR 0.44 µM
CB-rev1	GGTAAAGCCACAGTCTGCTCTA	RT-PCR 2.22 µM
pMXs-BamHI-InFusion	AGTTAATTAAG GATC c gcc	2nd/3rd PCR
CB-rev2	CTGTGCACCTCCTTCCCATTC	2nd PCR
CB-rev3	GTGGCCAGGCACACCAGTGT	Sequence/3rd PCR
