# Supplementary material for: Identification of TCR repertoires in functionally competent cytotoxic T cells cross-reactive to SARS-CoV-2
Source: Commun Biol. 2021 Dec 2;4:1365. doi: 10.1038/s42003-021-02885-6 (PMC8640030; doi:10.1038/s42003-021-02885-6)
Supplement: Supplementary file 2 — Description of Additional Supplementary Files [file 42003_2021_2885_MOESM2_ESM.pdf]

Description of Additional Supplementary Files

File name: Supplementary Data 1

Description: All source data of main figures and supplementary figures.
