# Supplementary material for: Identification of TCR repertoires in functionally competent cytotoxic T cells cross-reactive to SARS-CoV-2
Source: Commun Biol. 2021 Dec 2;4:1365. doi: 10.1038/s42003-021-02885-6 (PMC8640030; doi:10.1038/s42003-021-02885-6)
Supplement: Supplementary file 4 — Reporting Summary [file 42003_2021_2885_MOESM4_ESM.pdf]

nature research

Corresponding author(s): Shin-ichiro Fujii

Last updated by author(s): Nov 5, 2021

Reporting Summary

Nature Research wishes to improve the reproducibility of the work that we publish. This form provides structure for consistency and transparency in reporting. For further information on Nature Research policies, see our Editorial Policies and the Editorial Policy Checklist.

Statistics

For all statistical analyses, confirm that the following items are present in the figure legend, table legend, main text, or Methods section.

n/a	Confirmed	
☐	☒	The exact sample size (n) for each experimental group/condition, given as a discrete number and unit of measurement
☐	☒	A statement on whether measurements were taken from distinct samples or whether the same sample was measured repeatedly
☐	☒	The statistical test(s) used AND whether they are one- or two-sided<br>*Only common tests should be described solely by name; describe more complex techniques in the Methods section.*
☒	☐	A description of all covariates tested
☐	☒	A description of any assumptions or corrections, such as tests of normality and adjustment for multiple comparisons
☐	☒	A full description of the statistical parameters including central tendency (e.g. means) or other basic estimates (e.g. regression coefficient) AND variation (e.g. standard deviation) or associated estimates of uncertainty (e.g. confidence intervals)
☐	☒	For null hypothesis testing, the test statistic (e.g. F, t, r) with confidence intervals, effect sizes, degrees of freedom and P value noted<br>*Give P values as exact values whenever suitable.*
☒	☐	For Bayesian analysis, information on the choice of priors and Markov chain Monte Carlo settings
☒	☐	For hierarchical and complex designs, identification of the appropriate level for tests and full reporting of outcomes
☐	☒	Estimates of effect sizes (e.g. Cohen's d, Pearson's r), indicating how they were calculated

Our web collection on statistics for biologists contains articles on many of the points above.

Software and code

Policy information about availability of computer code

Data collection	BD FACSDivaTM Software v.8.0.1<br>COVID-19 information (https://www.who.int/emergencies/diseases/novel-coronavirus-2019)<br>Allele Frequency Net Database (AFND) (www.allelefrequencies.net)<br>ZOO/KAMO (Hirata et al.2019, Yamashita et al.2018)
Data analysis	NetMHC4.0 (http://www.cbs.dtu.dk/services/NetMHC/)<br>IEBD (https://www.iedb.org/)<br>Flow jo v10.3B2<br>IMGT/V-QUEST(http://www.imgt.org/IMGT_vquest/input)<br>StatMate V(Nihon 3B Scientific Inc.)<br>Phaser: (version 2.8.1/2.8.3)<br>Phenix (version 1.13_2998)<br>COOT (version 0.8.9.3-pre)<br>PyMOL (version 1.4.1)<br>ccp4i (version 6.5)

For manuscripts utilizing custom algorithms or software that are central to the research but not yet described in published literature, software must be made available to editors and reviewers. We strongly encourage code deposition in a community repository (e.g. GitHub). See the Nature Research guidelines for submitting code & software for further information.

Data

Policy information about availability of data

All manuscripts must include a data availability statement. This statement should provide the following information, where applicable:

- Accession codes, unique identifiers, or web links for publicly available datasets
- A list of figures that have associated raw data
- A description of any restrictions on data availability

The structures of HLA-A*24:02•peptide (CoV2, 229E, and HKU1) complexes were deposited in the RCSB Protein DataBank (PDB) under accession codes 7EJL, 7EJM, and 7EJN respectively. The single TCR seq data is being processed to upload to database. All source data underlying the graphs presented in the main figures and supplementary figures are available in Supplementary Data 1.

Field-specific reporting

Please select the one below that is the best fit for your research. If you are not sure, read the appropriate sections before making your selection.

☒ Life sciences ☐ Behavioural & social sciences ☐ Ecological, evolutionary & environmental sciences

For a reference copy of the document with all sections, see nature.com/documents/nr-reporting-summary-flat.pdf

Life sciences study design

All studies must disclose on these points even when the disclosure is negative.

Sample size	Sample sizes were based on maximal available sample sets. Sample size calculation was not determined prior to investigation. The reason for this study was in a novel with no previous information. We obtained PBMCs from HLA-A24 positive healthy volunteers in our institute and also them from Tokyo Red Cross (total n=21). HLA-A24 positive patients with hematological malignancies were recruited for donation of PBMCs in the National Hospital Organization Kumamoto Medical Center (total n=28).
Data exclusions	No data was excluded from the analyses except the limitation of cell numbers.
Replication	Samples analyzed in this study were from participants of a cohort study and samples were analyzed on individual study participants. Experiments did not include replicates as all participants and data points are unique. Some of the experiments used technical and/or biological replicates, and all attempts were successful.
Randomization	No randomization was performed.
Blinding	Blinding was not appropriate for this study of T cell responses in healthy volunteers and patients, with no associated therapeutic intervention.

Reporting for specific materials, systems and methods

We require information from authors about some types of materials, experimental systems and methods used in many studies. Here, indicate whether each material, system or method listed is relevant to your study. If you are not sure if a list item applies to your research, read the appropriate section before selecting a response.

Materials & experimental systems

n/a	Involved in the study	
☐	☒	Antibodies
☐	☒	Eukaryotic cell lines
☒	☐	Palaeontology and archaeology
☒	☐	Animals and other organisms
☐	☒	Human research participants
☒	☐	Clinical data
☒	☐	Dual use research of concern

Methods

n/a	Involved in the study	
☒	☐	ChIP-seq
☐	☒	Flow cytometry
☒	☐	MRI-based neuroimaging

Antibodies

Antibodies used

Antibodies used in this study are described fully in Supplementary Table 4.
Mouse anti-human CD3 PE/Cyanine7 clone: UCHT1; cat: 300420; Biolegend (1:500 dilution)
Mouse anti-human CD4 PerCP/Cy5.5 clone: OKT4; cat: 317428; Biolegend (1:100 dilution) Mouse
anti-human CD4 PerCP/Cy5.5 clone: RPA-T4; cat: 300530; Biolegend (1:100 dilution) Mouse anti-
human CD8a FITC clone: RPA-T8; cat: 301006; Biolegend (1:200 dilution) Mouse Anti-
Human CD8 BUV737 clone: SK1; cat: 564629; BD Biosciences (1:500 dilution) Mouse anti-
human CD69 APC clone: FN50; cat: 310904; Biolegend (1:400 dilution) Mouse anti-

	human CD107a BV421 clone: H4A3; cat: 328626; Biolegend (1:100 dilution)<br>Mouse anti-human CD107a Alexa Fluor 488 clone: H4A3; cat: 328626; Biolegend (1:40 dilution)<br>Rat anti-human IL-2 BV785 clone: MQ1-17H12; cat: 500348; Biolegend (1:100 dilution)<br>Rat Anti-Human and Viral IL-10 PE clone:JES3-9D7; cat: 559337; BD Biosciences (1:20 dilution) Mouse<br>anti-human TNF-α PE clone: MAb11; cat: 502909; Biolegend (1:200 dilution) Mouse<br>anti-human IFN-γ APC clone: B27; cat: 506510; Biolegend (1:100 dilution) Mouse<br>Anti-Human HLA-A24 FITC clone: 17A10; cat: K0208-4; MBL (1:100 dilution)
Validation	All antibodies were purchased from the above stated companies. Antibodies are well described and published elsewhere. Informations can be sought from the manufacures website under catalogue number.

Eukaryotic cell lines

Policy information about cell lines

Cell line source(s)	CIR cells expressing HLA-A*24:02 (A24/CIR) were provided by Dr. Masafumi Takiguchi (Kumamoto University). The SKW-3 (T-ALL) line was purchased from RIKEN BRC (Japan).
Authentication	A24/CIR has been routinely tested for HLA-A24 expression by our hands. With regard to SKW-3 cells, we verified that our cells lack TCRs and functionally work like T cells when transduced with T cell receptors and CD8AB.
Mycoplasma contamination	Cell lines were tested for mycoplasma and there is no contamination.
Commonly misidentified lines (See ICLAC register)	According to the International Cell Line Authentication Committee register (https://iclac.org), SKW-3 cells were misidentified as KE-37 cells.

Human research participants

Policy information about studies involving human research participants

Population characteristics	The information is provided accordingly in methods section and in supplementary Table 1 and 2.
Recruitment	Human peripheral blood were obtained from healthy volunteers in our institute or from Tokyo Red Cross, and patients with hematological malignancies in the National Hospital Organization Kumamoto Medical Center. All the donors were not infected with SARS-Cov-2.
Ethics oversight	This study was performed in compliance with the Declaration of Helsinki and approved by the ethic committee of RIKEN (H26-18(10)). Written informed consent was obtained from all participants.

Note that full information on the approval of the study protocol must also be provided in the manuscript.

Flow Cytometry

Plots

Confirm that:

- [x] The axis labels state the marker and fluorochrome used (e.g. CD4-FITC).
- [x] The axis scales are clearly visible. Include numbers along axes only for bottom left plot of group (a 'group' is an analysis of identical markers).
- [x] All plots are contour plots with outliers or pseudocolor plots.
- [x] A numerical value for number of cells or percentage (with statistics) is provided.

Methodology

Sample preparation	Whole blood were collected in EDTA tubes. The PBMCs were isolated using Ficoll.<br>Detail culture condition was described in the Methods section.
Instrument	BD LSR Fortessa X-20, BD FACS Canto , BD FACS ARIA III Cell Sorter
Software	Data was acquired with the Diva software (BD Bioscience) and analyzed with FlowJo v10.3B2 (BD Bioscience)
Cell population abundance	Cells populations were reported as a proportion of a specific population (% IFNγ+TNFα+ cells in CD8T etc).

Gating strategy	Lymphocytes were selected in the FSC/SSC gate. Single cell were selected. Viable cells were determined using Aqua live/dead (Invitrogen). Live CD3+ cells were divided in CD3+CD4+(CD4+ T cells) and CD3+CD8+ (CD8+ T cells). CD4+ T cells and CD8+ T cells were analyzed separately for production of indicated cytokines and expression of degranulation marker.

☐ Tick this box to confirm that a figure exemplifying the gating strategy is provided in the Supplementary Information.
